# A communal catalogue reveals Earth’s multiscale microbial diversity

**DOI:** 10.1038/nature24621

**Published:** 2017-11-01

**Authors:** Luke R. Thompson, Jon G. Sanders, Daniel McDonald, Amnon Amir, Joshua Ladau, Kenneth J. Locey, Robert J. Prill, Anupriya Tripathi, Sean M. Gibbons, Gail Ackermann, Jose A. Navas-Molina, Stefan Janssen, Evguenia Kopylova, Yoshiki Vázquez-Baeza, Antonio González, James T. Morton, Siavash Mirarab, Zhenjiang Zech Xu, Lingjing Jiang, Mohamed F. Haroon, Jad Kanbar, Qiyun Zhu, Se Jin Song, Tomasz Kosciolek, Nicholas A. Bokulich, Joshua Lefler, Colin J. Brislawn, Gregory Humphrey, Sarah M. Owens, Jarrad Hampton-Marcell, Donna Berg-Lyons, Valerie McKenzie, Noah Fierer, Jed A. Fuhrman, Aaron Clauset, Rick L. Stevens, Ashley Shade, Katherine S. Pollard, Kelly D. Goodwin, Janet K. Jansson, Jack A. Gilbert, Rob Knight, The Earth Microbiome Project Consortium, Jose L. Agosto Rivera, Lisa Al-Moosawi, John Alverdy, Katherine R. Amato, Jason Andras, Largus T. Angenent, Dionysios A. Antonopoulos, Amy Apprill, David Armitage, Kate Ballantine, Jirˇí Bárta, Julia K. Baum, Allison Berry, Ashish Bhatnagar, Monica Bhatnagar, Jennifer F. Biddle, Lucie Bittner, Bazartseren Boldgiv, Eric Bottos, Donal M. Boyer, Josephine Braun, William Brazelton, Francis Q. Brearley, Alexandra H. Campbell, J. Gregory Caporaso, Cesar Cardona, JoLynn Carroll, S. Craig Cary, Brenda B. Casper, Trevor C. Charles, Haiyan Chu, Danielle C. Claar, Robert G. Clark, Jonathan B. Clayton, Jose C. Clemente, Alyssa Cochran, Maureen L. Coleman, Gavin Collins, Rita R. Colwell, Mónica Contreras, Benjamin B. Crary, Simon Creer, Daniel A. Cristol, Byron C. Crump, Duoying Cui, Sarah E. Daly, Liliana Davalos, Russell D. Dawson, Jennifer Defazio, Frédéric Delsuc, Hebe M. Dionisi, Maria Gloria Dominguez-Bello, Robin Dowell, Eric A. Dubinsky, Peter O. Dunn, Danilo Ercolini, Robert E. Espinoza, Vanessa Ezenwa, Nathalie Fenner, Helen S. Findlay, Irma D. Fleming, Vincenzo Fogliano, Anna Forsman, Chris Freeman, Elliot S. Friedman, Giancarlo Galindo, Liza Garcia, Maria Alexandra Garcia-Amado, David Garshelis, Robin B. Gasser, Gunnar Gerdts, Molly K. Gibson, Isaac Gifford, Ryan T. Gill, Tugrul Giray, Antje Gittel, Peter Golyshin, Donglai Gong, Hans-Peter Grossart, Kristina Guyton, Sarah-Jane Haig, Vanessa Hale, Ross Stephen Hall, Steven J. Hallam, Kim M. Handley, Nur A. Hasan, Shane R. Haydon, Jonathan E. Hickman, Glida Hidalgo, Kirsten S. Hofmockel, Jeff Hooker, Stefan Hulth, Jenni Hultman, Embriette Hyde, Juan Diego Ibáñez-Álamo, Julie D. Jastrow, Aaron R. Jex, L. Scott Johnson, Eric R. Johnston, Stephen Joseph, Stephanie D. Jurburg, Diogo Jurelevicius, Anders Karlsson, Roger Karlsson, Seth Kauppinen, Colleen T. E. Kellogg, Suzanne J. Kennedy, Lee J. Kerkhof, Gary M. King, George W. Kling, Anson V. Koehler, Monika Krezalek, Jordan Kueneman, Regina Lamendella, Emily M. Landon, Kelly Lane-deGraaf, Julie LaRoche, Peter Larsen, Bonnie Laverock, Simon Lax, Miguel Lentino, Iris I. Levin, Pierre Liancourt, Wenju Liang, Alexandra M. Linz, David A. Lipson, Yongqin Liu, Manuel E. Lladser, Mariana Lozada, Catherine M. Spirito, Walter P. MacCormack, Aurora MacRae-Crerar, Magda Magris, Antonio M. Martín-Platero, Manuel Martín-Vivaldi, L. Margarita Martínez, Manuel Martínez-Bueno, Ezequiel M. Marzinelli, Olivia U. Mason, Gregory D. Mayer, Jamie M. McDevitt-Irwin, James E. McDonald, Krista L. McGuire, Katherine D. McMahon, Ryan McMinds, Mónica Medina, Joseph R. Mendelson, Jessica L. Metcalf, Folker Meyer, Fabian Michelangeli, Kim Miller, David A. Mills, Jeremiah Minich, Stefano Mocali, Lucas Moitinho-Silva, Anni Moore, Rachael M. Morgan-Kiss, Paul Munroe, David Myrold, Josh D. Neufeld, Yingying Ni, Graeme W. Nicol, Shaun Nielsen, Jozef I. Nissimov, Kefeng Niu, Matthew J. Nolan, Karen Noyce, Sarah L. O’Brien, Noriko Okamoto, Ludovic Orlando, Yadira Ortiz Castellano, Olayinka Osuolale, Wyatt Oswald, Jacob Parnell, Juan M. Peralta-Sánchez, Peter Petraitis, Catherine Pfister, Elizabeth Pilon-Smits, Paola Piombino, Stephen B. Pointing, F. Joseph Pollock, Caitlin Potter, Bharath Prithiviraj, Christopher Quince, Asha Rani, Ravi Ranjan, Subramanya Rao, Andrew P. Rees, Miles Richardson, Ulf Riebesell, Carol Robinson, Karl J. Rockne, Selena Marie Rodriguezl, Forest Rohwer, Wayne Roundstone, Rebecca J. Safran, Naseer Sangwan, Virginia Sanz, Matthew Schrenk, Mark D. Schrenzel, Nicole M. Scott, Rita L. Seger, Andaine Seguin-Orlando, Lucy Seldin, Lauren M. Seyler, Baddr Shakhsheer, Gabriela M. Sheets, Congcong Shen, Yu Shi, Hakdong Shin, Benjamin D. Shogan, Dave Shutler, Jeffrey Siegel, Steve Simmons, Sara Sjöling, Daniel P. Smith, Juan J. Soler, Martin Sperling, Peter D. Steinberg, Brent Stephens, Melita A. Stevens, Safiyh Taghavi, Vera Tai, Karen Tait, Chia L. Tan, Neslihan Tas¸, D. Lee Taylor, Torsten Thomas, Ina Timling, Benjamin L. Turner, Tim Urich, Luke K. Ursell, Daniel van der Lelie, William Van Treuren, Lukas van Zwieten, Daniela Vargas-Robles, Rebecca Vega Thurber, Paola Vitaglione, Donald A. Walker, William A. Walters, Shi Wang, Tao Wang, Tom Weaver, Nicole S. Webster, Beck Wehrle, Pamela Weisenhorn, Sophie Weiss, Jeffrey J. Werner, Kristin West, Andrew Whitehead, Susan R. Whitehead, Linda A. Whittingham, Eske Willerslev, Allison E. Williams, Stephen A. Wood, Douglas C. Woodhams, Yeqin Yang, Jesse Zaneveld, Iratxe Zarraonaindia, Qikun Zhang, Hongxia Zhao

**Affiliations:** 1 Department of Pediatrics, University of California San Diego, La Jolla, California USA; 2 Department of Biological Sciences and Northern Gulf Institute, University of Southern Mississippi, Hattiesburg, Mississippi USA; 3 Ocean Chemistry and Ecosystems Division, Atlantic Oceanographic and Meteorological Laboratory, National Oceanic and Atmospheric Administration, stationed at Southwest Fisheries Science Center, La Jolla, California USA; 4 The Gladstone Institutes and University of California San Francisco, San Francisco, California USA; 5 Department of Biology, Indiana University, Bloomington, Indiana USA; 6 Industrial and Applied Genomics, IBM Almaden Research Center, San Jose, California USA; 7 Division of Biological Sciences, University of California San Diego, La Jolla, California USA; 8 Skaggs School of Pharmacy, University of California San Diego, La Jolla, California USA; 9 Department of Biological Engineering, Massachusetts Institute of Technology, Cambridge, Massachusetts USA; 10 The Broad Institute of MIT and Harvard, Cambridge, Massachusetts USA; 11 Department of Computer Science and Engineering, University of California San Diego, La Jolla, California USA; 12 Department of Electrical and Computer Engineering, University of California San Diego, La Jolla, California USA; 13 Department of Family Medicine and Public Health, University of California San Diego, La Jolla, California USA; 14 Department of Organismic and Evolutionary Biology, Harvard University, Cambridge, Massachusetts USA; 15 Pathogen and Microbiome Institute, Northern Arizona University, Flagstaff, Arizona USA; 16 Earth and Biological Sciences Directorate, Pacific Northwest National Laboratory, Richland, Washington USA; 17 Biosciences Division, Argonne National Laboratory, Argonne, Illinois USA; 18 Department of Biological Sciences, University of Illinois at Chicago, Chicago, Illinois USA; 19 BioFrontiers Institute, University of Colorado, Boulder, Colorado USA; 20 Department of Ecology and Evolutionary Biology, University of Colorado, Boulder, Colorado USA; 21 Cooperative Institute for Research in Environmental Sciences, University of Colorado, Boulder, Colorado USA; 22 Department of Biological Sciences, University of Southern California, Los Angeles, California USA; 23 Department of Computer Science, University of Colorado, Boulder, Colorado USA; 24 Computing, Environment and Life Sciences, Argonne National Laboratory, Argonne, Illinois USA; 25 Department of Computer Science, University of Chicago, Chicago, Illinois USA; 26 Department of Microbiology and Molecular Genetics, Michigan State University, East Lansing, Michigan USA; 27 Department of Plant, Soil and Microbial Sciences, Michigan State University, East Lansing, Michigan USA; 28 Program in Ecology, Evolutionary Biology and Behavior, Michigan State University, East Lansing, Michigan USA; 29 Department of Surgery, University of Chicago, Chicago, Illinois USA; 30 Center for Microbiome Innovation, University of California San Diego, La Jolla, California USA; 31 University of Puerto Rico, San Juan, Puerto Rico USA; 32 Plymouth Marine Laboratory, Plymouth, England UK; 33 University of Chicago, Chicago, Illinois USA; 34 Northwestern University, Evanston, Illinois USA; 35 Mount Holyoke College, South Hadley, Massachusetts USA; 36 Cornell University, Ithaca, New York, USA; 37 University of Tübingen, Tübingen, Germany; 38 Argonne National Laboratory, Argonne, Illinois USA; 39 Woods Hole Oceanographic Institution, Woods Hole, Massachusetts USA; 40 University of California Berkeley, Berkeley, California USA; 41 University of Notre Dame, South Bend, Indiana USA; 42 University of South Bohemia, Cˇeské Bude˘jovice, Czech Republic; 43 University of Victoria, Victoria, British Columbia Canada; 44 University of California Davis, Davis, California USA; 45 Maharshi Dayanand Saraswati University, Ajmer, India; 46 University of Delaware, Newark, Delaware USA; 47 Université Pierre et Marie Curie, Evolution Paris Seine, Paris, France; 48 National University of Mongolia, Ulaanbaatar, Mongolia; 49 Pacific Northwest National Laboratory, Richland, Washington USA; 50 Wildlife Conservation Society and Bronx Zoo, New York, New York USA; 51 San Diego Zoo Institute for Conservation Research, Escondido, California USA; 52 University of Utah, Salt Lake City, Utah USA; 53 Manchester Metropolitan University, Manchester, UK; 54 University of New South Wales, Sydney, New South Wales Australia; 55 Northern Arizona University, Flagstaff, Arizona USA; 56 UiT-The Arctic University of Norway, Tromsø, Norway; 57 University of Waikato, Hamilton, New Zealand; 58 University of Pennsylvania, Philadelphia, Pennsylvania USA; 59 University of Waterloo, Waterloo, Ontario Canada; 60 Institute of Soil Science, Chinese Academy of Sciences, Nanjing, China; 61 Environment and Climate Change Canada, Saskatoon, Canada; 62 University of Minnesota, Saint Paul, Minnesota USA; 63 GreenViet Biodiversity Conservation Center, Da Nang, Viet Nam; 64 Icahn School of Medicine at Mount Sinai, New York, New York USA; 65 Colorado State University, Fort Collins, Colorado USA; 66 National University of Ireland, Galway, Ireland; 67 University of Maryland, College Park, Maryland USA; 68 Instituto Venezolano de Investigaciones Cientificas (IVIC), Venezuela; 69 University of Wisconsin, Madison, Wisconsin USA; 70 Bangor University, Bangor, Gwynedd UK; 71 College of William and Mary, Williamsburg, Virginia USA; 72 Oregon State University, Corvallis, Oregon USA; 73 Beijing Zoo, Beijing, China; 74 Purdue University, West Lafayette, Indiana USA; 75 Stony Brook University, Stony Brook, New York USA; 76 University of Northern British Columbia, Prince George, British, Columbia Canada; 77 Université de Montpellier, CNRS, Montpellier France; 78 Centro para el Estudio de Sistemas Marinos (CESIMAR-CONICET), CCT CENPAT, Puerto Madryn, Chubut Argentina; 79 New York University, New York, New York USA; 80 University of Colorado, Boulder, Colorado USA; 81 Lawrence Berkeley National Laboratory, Berkeley, California USA; 82 University of Wisconsin, Milwaukee, Wisconsin USA; 83 University of Naples Federico II, Naples, Italy; 84 California State University, Northridge, California USA; 85 University of Georgia, Athens, Georgia USA; 86 Vanderbilt University, Nashville, Tennessee USA; 87 University of Central Florida, Orlando, Florida USA; 88 Minnesota Department of Natural Resources, St. Paul, Minnesota USA; 89 University of Melbourne, Melbourne, Victoria Australia; 90 Huazhong Agricultural University, Wuhan, Hubei China; 91 Alfred Wegener Institute, Bremerhaven, Germany; 92 Washington University, St. Louis, Missouri USA; 93 Aarhus University, Aarhus, Denmark; 94 University of Bergen, Bergen, Norway; 95 Virginia Institute of Marine Science, Gloucester Point, Virginia USA; 96 Leibniz Institute for Freshwater Ecology and Inland Fisheries, Stechlin, Germany; 97 Potsdam University, Potsdam, Germany; 98 University of Michigan, Ann Arbor, Michigan USA; 99 The Ohio State University College of Veterinary Medicine, Columbus, Ohio USA; 100 University of British Columbia, Vancouver, British, Columbia Canada; 101 ECOSCOPE Training Program, Vancouver, British Columbia Canada; 102 University of Auckland, Auckland, New Zealand; 103 Melbourne Water Corporation, Melbourne, Victoria Australia; 104 Columbia University, New York, New York USA; 105 Amazonic Center for Research and Control of Tropical Diseases (CAICET), Puerto Ayacucho, Amazonas Venezuela; 106 Iowa State University, Ames, Iowa USA; 107 Chief Dull Knife College, Lame Deer, Montana USA; 108 University of Gothenburg, Gothenburg, Sweden; 109 University of Helsinki, Helsinki, Finland; 110 University of California San Diego, La Jolla, California USA; 111 University of Groningen, Groningen, The Netherlands; 112 The Walter and Eliza Hall Institute of Medical Research, Parkville, Victoria Australia; 113 Towson University, Towson, Maryland USA; 114 Georgia Institute of Technology, Atlanta, Georgia USA; 115 Wageningen University and Research, Wageningen, Netherlands; 116 Universidade Federal do Rio de Janeiro, Rio de Janeiro, Brazil; 117 Nanoxis Consulting AB, Gothenburg, Sweden; 118 Bio-Path Holdings, Inc., Texas, Bellaire USA; 119 Rutgers University, New Brunswick, New Jersey USA; 120 Louisiana State University, Baton Rouge, Louisiana USA; 121 Smithsonian Tropical Research Institute, Balboa, Ancon Panama; 122 Juniata College, Huntingdon, Pennsylvania USA; 123 Fontbonne University, St. Louis, Missouri USA; 124 Dalhousie University, Halifax, Nova Scotia Canada; 125 University of Technology, Sydney, New South Wales Australia; 126 Colección Ornitologica W. H. Phelps, Caracas, Venezuela; 127 Institute of Botany, Czech Academy of Sciences, Dukelská, Trebon Czech Republic; 128 Institute of Applied Ecology, Chinese Academy of Sciences, Shenyang, China; 129 San Diego State University, San Diego, California USA; 130 Institute of Tibetan Plateau Research, Chinese Academy of Sciences, Beijing, China; 131 Universidad de Buenos Aires, Ciudad Autónoma de, Buenos Aires Argentina; 132 Instituto Antártico Argentino, Buenos Aires, Argentina; 133 University of Granada, Granada, Spain; 134 Florida State University, Tallahassee, Florida USA; 135 Texas Tech University, Lubbock, Texas USA; 136 Stanford University, Stanford, California USA; 137 University of Oregon, Eugene, Oregon USA; 138 Pennsylvania State University, State College, Pennsylvania USA; 139 Zoo Atlanta, Atlanta, Georgia USA; 140 Ohio University, Athens, Ohio USA; 141 CREA - Centro Agricoltura e Ambiente (CREA-AA), Florence, Italy; 142 Morningside College, Sioux City, Iowa USA; 143 Miami University, Oxford, Ohio USA; 144 Université de Lyon, Lyon, France; 145 Fanjingshan National Nature Reserve Administration, Tongren, China; 146 University of Turin, Turin, Italy; 147 Natural History Museum of Denmark, Copenhagen, Denmark; 148 Université de Toulouse, Université Paul Sabatier, Toulouse, France; 149 Elizade University, Ilara-Mokin, Ondo State Nigeria; 150 Emerson College, Boston, Massachusetts USA; 151 Novozymes North America Inc., Raleigh-Durham, North Carolina USA; 152 Auckland University of Technology, Auckland, New Zealand; 153 City University of New York, New York, New York USA; 154 University of Warwick, Coventry, UK; 155 University of Illinois, Chicago, Illinois USA; 156 The Hong Kong Polytechnic University, Hong Kong, China; 157 GEOMAR Helmholtz Center for Ocean Research Kiel, Kiel, Germany; 158 University of East Anglia, Norwich, UK; 159 Department of Environmental Protection and Natural Resources, Northern Cheyenne Tribe, Lame Deer, Montana USA; 160 Michigan State University, East Lansing, Michigan USA; 161 Hybla Valley Veterinary Hospital, Alexandria, Virginia USA; 162 Biota Technology Inc., San Diego, California USA; 163 University of Maine, Orono, Maine USA; 164 University of Copenhagen, Copenhagen, Denmark; 165 Emory University, Atlanta, Georgia USA; 166 Sejong University, Seoul, South Korea; 167 Acadia University, Wolfville, Nova Scotia Canada; 168 University of Toronto, Toronto, Ontario Canada; 169 Independent Ornithologist, Merced, California USA; 170 Södertörn University, Huddinge, Sweden; 171 Baylor College of Medicine, Houston, Texas USA; 172 Estación Experimental de Zonas Áridas (EEZA-CSIC), Almería, Spain; 173 Illinois Institute of Technology, Chicago, Illinois USA; 174 Gusto Global LLC, Charlotte, North Carolina USA; 175 Western University, London, Ontario Canada; 176 LVDI International, San Marcos, California USA; 177 University of New Mexico, Albuquerque, New Mexico USA; 178 University of Alaska, Fairbanks, Alaska USA; 179 University of Vienna, Vienna, Austria; 180 Max Planck Institute for Developmental Biology, Tübingen, Germany; 181 Australian Institute of Marine Science, Townsville, Queensland Australia; 182 University of California Irvine, Irvine, California USA; 183 State University of New York, Cortland, New York USA; 184 DOCS Global, Research Triangle Park, North Carolina, USA; 185 Virginia Polytechnic Institute and State University, Blacksburg, Virginia USA; 186 Yale University, New Haven, Connecticut USA; 187 University of Massachusetts Boston, Boston, Massachusetts USA; 188 Tongren University, Tongren, Guizhou China; 189 University of Washington Bothell, Bothell, Washington USA; 190 University of the Basque Country (UPV/EHU), Leioa, Spain; 191 Zhejiang Institute of Microbiology, Hangzhou, Zhejiang China

**Keywords:** Environmental microbiology, Microbiome

## Abstract

Our growing awareness of the microbial world’s importance and diversity contrasts starkly with our limited understanding of its fundamental structure. Despite recent advances in DNA sequencing, a lack of standardized protocols and common analytical frameworks impedes comparisons among studies, hindering the development of global inferences about microbial life on Earth. Here we present a meta-analysis of microbial community samples collected by hundreds of researchers for the Earth Microbiome Project. Coordinated protocols and new analytical methods, particularly the use of exact sequences instead of clustered operational taxonomic units, enable bacterial and archaeal ribosomal RNA gene sequences to be followed across multiple studies and allow us to explore patterns of diversity at an unprecedented scale. The result is both a reference database giving global context to DNA sequence data and a framework for incorporating data from future studies, fostering increasingly complete characterization of Earth’s microbial diversity.

**Supplementary information:**

The online version of this article (doi:10.1038/nature24621) contains supplementary material, which is available to authorized users.

## Abstract (web summary)

As phase 1 of the Earth Microbiome Project, analysis of 16S ribosomal RNA sequences from more than 27,000 environmental samples delivers a global picture of the basic structure and drivers of microbial distribution.

**Supplementary information:**

The online version of this article (doi:10.1038/nature24621) contains supplementary material, which is available to authorized users.

## Earth's microbial diversity

In this meta-analysis of studies from the Earth Microbiome Project, Luke Thompson *et al*. have developed a standardized collection, curation and analysis pipeline to examine 16S ribosomal RNA (rRNA) tag sequences of more than 27,000 samples (representing soil, water, animal-associated and plant-associated habitats), providing global context for the basic structure and drivers of bacterial and archaeal distribution. The work not only provides an important reference database and framework for future studies in microbial ecology, but also reveals patterns in community composition as well as the global distribution of particular organisms. These findings provide a deeper understanding of dispersal and niche colonization.

**Supplementary information:**

The online version of this article (doi:10.1038/nature24621) contains supplementary material, which is available to authorized users.

## Main

A primary aim of microbial ecology is to determine patterns and drivers of community distribution, interaction, and assembly amidst complexity and uncertainty. Microbial community composition has been shown to change across gradients of environment, geographic distance, salinity, temperature, oxygen, nutrients, pH, day length, and biotic factors^1,2,3,4,5,6^. These patterns have been identified mostly by focusing on one sample type and region at a time, with insights extrapolated across environments and geography to produce generalized principles. To assess how microbes are distributed across environments globally—or whether microbial community dynamics follow fundamental ecological ‘laws’ at a planetary scale—requires either a massive monolithic cross-environment survey or a practical methodology for coordinating many independent surveys. New studies of microbial environments are rapidly accumulating; however, our ability to extract meaningful information from across datasets is outstripped by the rate of data generation. Previous meta-analyses have suggested robust general trends in community composition, including the importance of salinity^1^ and animal association^2^. These findings, although derived from relatively small and uncontrolled sample sets, support the utility of meta-analysis to reveal basic patterns of microbial diversity and suggest that a scalable and accessible analytical framework is needed.

The Earth Microbiome Project (EMP, http://www.earthmicrobiome.org) was founded in 2010 to sample the Earth’s microbial communities at an unprecedented scale in order to advance our understanding of the organizing biogeographic principles that govern microbial community structure^7,8^. We recognized that open and collaborative science, including scientific crowdsourcing and standardized methods^8^, would help to reduce technical variation among individual studies, which can overwhelm biological variation and make general trends difficult to detect^9^. Comprising around 100 studies, over half of which have yielded peer-reviewed publications (Supplementary Table 1), the EMP has now dwarfed by 100-fold the sampling and sequencing depth of earlier meta-analysis efforts^1,2^; concurrently, powerful analysis tools have been developed, opening a new and larger window into the distribution of microbial diversity on Earth. In establishing a scalable framework to catalogue microbiota globally, we provide both a resource for the exploration of myriad questions and a starting point for the guided acquisition of new data to answer them. As an example of using this tool, we present a meta-analysis of the EMP archive, tracking individual sequences across diverse samples and studies with standardized environmental descriptors, investigating large-scale ecological patterns, and exploring key hypotheses in ecological theory to serve as seeds for future research.

## A standardized and scalable approach

**Figure 1:**
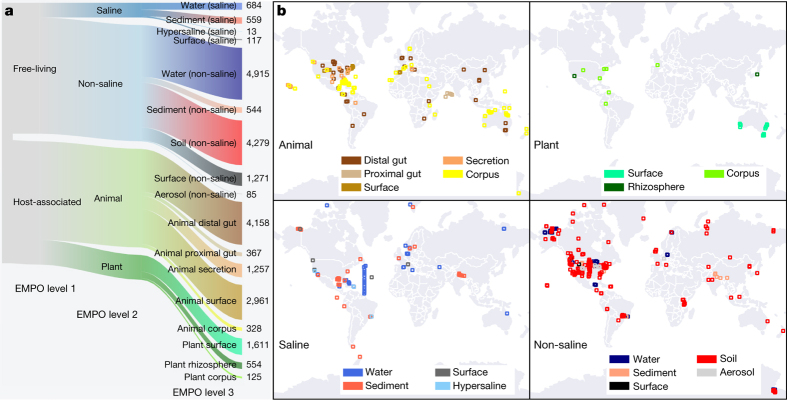
Environment type and provenance of samples. **a**, The EMP ontology (EMPO) classifies microbial environments (level 3) as free-living or host-associated (level 1) and saline or non-saline (if free-living) or animal or plant (if host-associated) (level 2). The number out of 23,828 samples in the QC-filtered subset in each environment is provided. EMPO is described with examples at http://www.earthmicrobiome.org/protocols-and-standards/empo. **b**, Global scope of sample provenance: samples come from 7 continents, 43 countries, 21 biomes (ENVO), 92 environmental features (ENVO), and 17 environments (EMPO). PowerPoint slide Source data

**Extended Data Figure 1:**
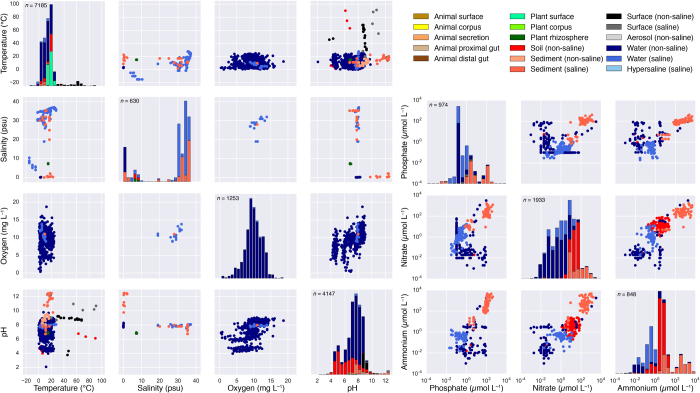
Physicochemical properties of the EMP samples. Pairwise scatter plots of available physicochemical metadata are shown for temperature, salinity, oxygen, and pH, and for phosphate, nitrate, and ammonium. Histograms for each factor are also shown; the number (*n*) of samples having data for each factor is provided at the top of each histogram. Samples are coloured by environment, and only QC-filtered samples are included. In sample metadata files, environmental factors are named in our recommended format, with analyte name and units combined in the metadata field name.

The EMP solicited the global scientific community for environmental samples and associated metadata spanning diverse environments and capturing spatial, temporal, and/or physicochemical covariation. The first 27,751 samples from 97 independent studies (Supplementary Table 1) represent diverse environment types (Fig. 1a), geographies (Fig. 1b), and chemistries (Extended Data Fig. 1). The EMP encompasses studies of bacterial, archaeal, and eukaryotic microbial diversity. The analysis here focuses exclusively on the bacterial and archaeal components of the overall database (for concision, use of ‘microbial’ will hereafter refer to bacteria and archaea only). Associated metadata included environment type, location information, host taxonomy (if relevant), and physicochemical measurements (Supplementary Table 2). Physicochemical measurements were made *in situ* at the time of sampling. Investigators were encouraged to measure temperature and pH at minimum. Salinity, oxygen, and inorganic nutrients were measured when possible, and investigators collected additional metadata pertinent to their particular investigations.

Metadata were required to conform to the Genomic Standards Consortium’s MIxS and Environment Ontology (ENVO) standards^10,11^. We also used a light-weight application ontology built on top of ENVO: the EMP Ontology (EMPO) of microbial environments. EMPO was tailored to capture two major environmental axes along which microbial beta-diversity has been shown to orient: host association and salinity^1,2^. We indexed the classes in this application ontology (Fig. 1a) as levels of a structured categorical variable to classify EMP samples as host-associated or free-living (level 1). Samples were categorized within those classes as animal-associated versus plant-associated or saline versus non-saline, respectively (level 2). A finer level (level 3) was then assigned that satisfied the degree of environment granularity sought for this meta-analysis (for example, sediment (saline), plant rhizosphere, or animal distal gut). We expect EMPO to evolve as new studies and sample types are added to the EMP and as additional patterns of beta-diversity are revealed.

**Extended Data Figure 2:**
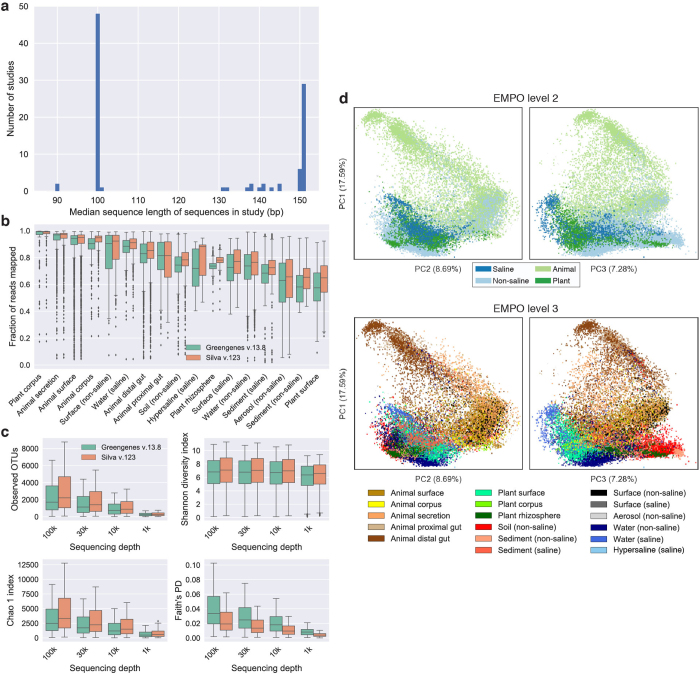
Sequence length, database effects, and beta-diversity patterns. **a**, Median sequence length per study after quality trimming. Original EMP studies used 90-bp reads, which were replaced by 100-bp reads for the majority of studies, and have since been replaced by 150–151-bp reads. For most analyses presented in this manuscript, we used the Deblur algorithm and trimmed tag sequences to 90 bp. This allowed inclusion of older studies with shorter read lengths. **b**, Comparison of Greengenes and SILVA rRNA databases for reference-based OTU picking. Fraction of reads in *n* = 23,828 biologically independent samples—separated by environment (per-environment *n* shown in Fig. 1a)—mapping to Greengenes 13.8 and SILVA 123 (97% identity OTUs) with closed-reference OTU picking. Boxplots show median, IQR, and 1.5 × IQR (with outliers). The fraction of reads mapping was similar between Greengenes and SILVA in each environment but slightly higher with SILVA for every environment. **c**, Alpha-diversity in closed-reference OTUs picked against Greengenes 13.8 and SILVA 123, with sequences rarefied to 100,000, 30,000, 10,000, and 1,000 sequences per sample, displayed as boxplots showing median, IQR, and 1.5 × IQR (with outliers). The sample set for all calculations contained *n* = 4,667 biologically independent samples having at least 100,000 observations in both Greengenes and SILVA OTU tables. Alpha-diversity metrics were higher with SILVA closed-reference OTU picking than with Greengenes. **d**, Beta-diversity among all EMP samples using principal coordinates analysis (PCA) of weighted UniFrac distance. Principal coordinates PC1 versus PC2 and PC1 versus PC3 are shown coloured by EMPO levels 2 and 3. As with unweighted UniFrac distance (Fig. 2c), clustering of samples using weighted UniFrac distance could be explained largely by environment.

We surveyed bacterial and archaeal diversity using amplicon sequencing of the 16S rRNA gene, a common taxonomic marker for bacteria and archaea^12^ that remains a valuable tool for microbial ecology despite the introduction of whole-genome methods (for example, shotgun metagenomics) that capture gene-level functional diversity^13^. DNA was extracted from samples using the MO BIO PowerSoil DNA extraction kit, PCR-amplified, and sequenced on the Illumina platform. Standardized DNA extraction was chosen to minimize the potential bias introduced by different extraction methodologies; however, extraction efficiency may also be subject to interactions between sample type and cell type, and thus extraction effects should be considered as a possible confounding factor in interpreting results. We amplified the 16S rRNA gene (V4 region) using primers^14^ shown to recover sequences from most bacterial taxa and many archaea^15^. We note that these primers may miss newly discovered phyla with alternative ribosomal gene structures^16^, and subsequent modifications not used here have shown improved efficiency with certain clades of Alphaproteobacteria and Archaea^17,18,19^. We generated sequence reads of 90–151 base pairs (bp) (Extended Data Fig. 2a, Supplementary Table 1), totaling 2.2 billion sequences, an average of 80,000 sequences per sample.

**Extended Data Figure 3:**
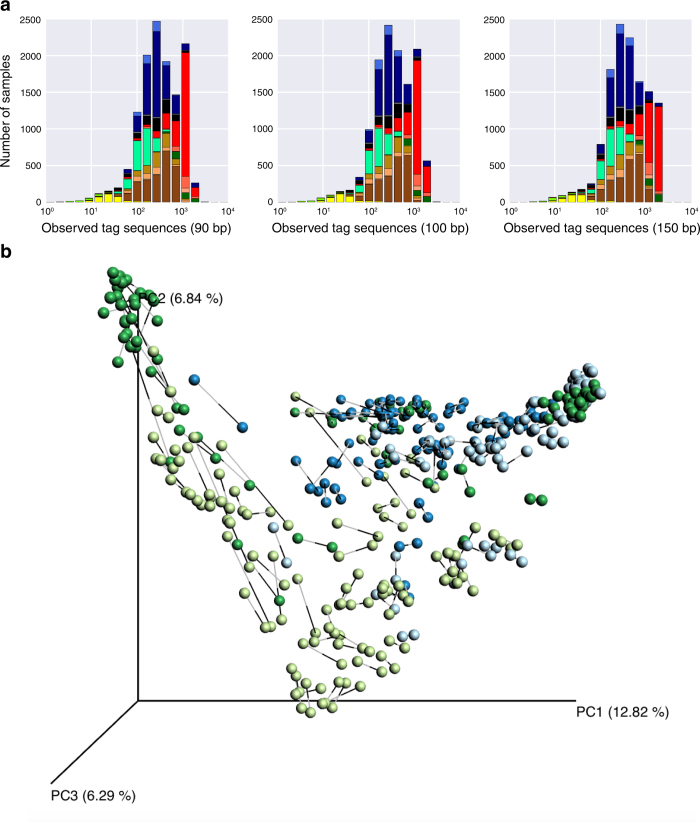
Sequence length effects on observed diversity patterns. The effect of trimming from 150 bp (the approximate starting length of some sequences) to 90 bp (the trimmed length of all sequences in this meta-analysis) was investigated by comparing alpha- and beta-diversity patterns. All samples, at each sequence length, were rarefied to 5,000 sequences per sample. **a**, Alpha-diversity distributions of *n* = 12,538 biologically independent samples displayed as histograms of observed tag sequences coloured by environment (EMPO level 3). Among these samples with sequence length ≥ 150 bp, the distributions are largely preserved when trimming from 150 to 100 to 90 bp. **b**, Procrustes goodness-of-fit between the 90-bp (grey lines) and 150-bp (black lines) Deblur principal coordinates (unweighted UniFrac distance) for *n* = 200 randomly chosen samples coloured by environment (EMPO level 2). Beta-diversity patterns between the two sequence lengths are similar.

Sequence analysis and taxonomic profiling were done initially using the common approach of assigning sequences to operational taxonomic units (OTUs) clustered by sequence similarity to existing rRNA databases^14,20^. While this approach was useful for certain analyses, for many sample types, especially plant-associated and free-living communities, one-third of reads or more could not be mapped to existing rRNA databases (Extended Data Fig. 2b). We therefore used a reference-free method, Deblur^21^, to remove suspected error sequences and provide single-nucleotide resolution ‘sub-OTUs’, also known as ‘amplicon sequence variants’^22^, here called ‘tag sequences’ or simply ‘sequences’. Because Deblur tag sequences for a given meta-analysis must be the same length in each sample, and some of the EMP studies have read lengths of 90 bp, we trimmed all sequences to 90 bp for this meta-analysis. We verified that the patterns presented here were not adversely affected by trimming the sequences (Extended Data Fig. 3). As we show, 90-bp sequences were sufficiently long to reveal detailed patterns of community structure. Because exact sequences are stable identifiers, unlike OTUs, they can be compared to any 16S rRNA or genomic database now and in the future, thereby promoting reusability^22^.

## Microbial ecology without OTU clustering

**Extended Data Figure 4:**
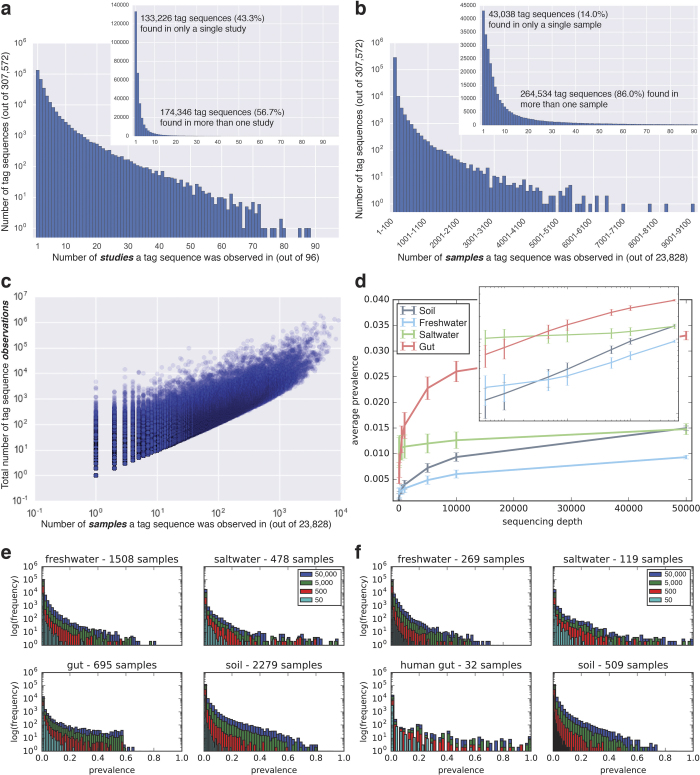
Tag sequence prevalence patterns. Note that for this meta-analysis, the input observation table was filtered to keep only tag sequences with at least 25 observations total over all samples and then rarefied to 5,000 observations per sample. **a**, Per-study endemism visualized as a histogram of tag sequences binned by the number of studies in which they are observed (right: linear scale; left: log scale). **b**, Per-sample endemism visualized as a histogram of tag sequences binned by the number of samples in which they are observed (right: sample counts up to 92 samples and the number of tag sequences in linear scale; left: all tag sequences with bin widths of 100 samples and number of tag sequences in log scale). **c**, Abundance (total observations in rarefied table) versus prevalence (number of samples observed in) of *n* = 307,572 tag sequences. Both axes are log scale. The most prevalent tag sequences were also the most abundant. **d**, Prevalence as a function of sequencing depth in *n* = 2,279 soil, *n* = 478 saltwater, *n* = 1,508 freshwater, and *n* = 695 animal distal gut samples having at least 50,000 sequences per sample. Shown are the average and s.d. of mean prevalence across triplicate rarefied subsamples of 50, 100, 500, 1,000, 5,000, 10,000, and 50,000 sequences per sample. Average prevalence increases with sequencing depth, and the straight-line relationship on the log–log axis is suggestive of a power law. **e**, Histograms of tag sequence prevalences at each sampling depth. The histograms show the distribution moving towards higher prevalences with increasing sequencing depth. Gut data lacked tag sequence prevalences > 0.7 owing to the inclusion of very different host species; see **f**. **f**, Histograms as in **e** but on a subset of the observation tables where 30 samples were randomly sampled from each study. Restricting to human gut samples only, the full range of prevalences found in the other environments is observed.

While earlier large-scale 16S rRNA amplicon studies adopted OTU clustering approaches in part out of concern that erroneous reads would dominate diversity assessments^23^, patterns of prevalence (presence–absence) in our results suggest that Deblur error removal produced ecologically informative sequences without clustering. After rarefying to 5,000 sequences per sample, a total of 307,572 unique sequences were contained in the 96 studies and 23,828 samples of the ‘QC-filtered’ Deblur 90-bp observation table. Among studies, more than half (57%) of all obtained sequences were observed in two or more studies, but only 5% were observed in more than ten studies; the most prevalent sequence was found in 88 of 96 studies (Extended Data Fig. 4a). Among samples, although most sequences (86%) were observed in two or more samples, only 7% were observed in more than 100 samples (Extended Data Fig. 4b). As expected, the most prevalent sequences were also the most abundant (Extended Data Fig. 4c).

Our analyses were carried out using a modest sequencing depth of 5,000 observations per sample after Deblur and rarefaction. To investigate how prevalence estimates were affected by sequencing depth, we focused on four major environment types for which we had the greatest number of samples with more than 50,000 observations (soil, saltwater, freshwater, and animal distal gut). The relationship between average tag sequence prevalence and sequencing depth differed among these environments (Extended Data Fig. 4d) but was generally positive, suggesting that our global analysis underestimated true prevalence. Animal-associated microbiomes were a notable exception, with an upper bound on prevalence apparently imposed by host specificity when all host species were considered (Extended Data Fig. 4e); this bound disappeared when considering only human-derived samples (Extended Data Fig. 4f). Although contamination remains an issue in microbiome studies^24^, most of the very highly abundant and prevalent sequences here had higher mean relative abundances among samples than among no-template controls (Supplementary Table 3), suggesting that they did not originate from reagents.

Matches between our sequences and existing 16S rRNA gene reference databases highlight the novelty captured by the EMP. Exact matches to 46% of Greengenes^25^ and 45% of SILVA^26^ rRNA gene databases were found in our dataset, indicating that we ‘recaptured’ nearly half of the reference sequence diversity with just under 100 environmental surveys. These matches accounted for 10% and 13%, respectively, of the tag sequences in our dataset, indicating that large swathes of microbial community diversity are not yet captured in full-length sequence databases. The failure of many sequences to be mapped in reference-based alignments to Greengenes and SILVA 97% identity OTUs (Extended Data Fig. 2b) supports this observation.

## Patterns of diversity reflect environment

**Figure 2:**
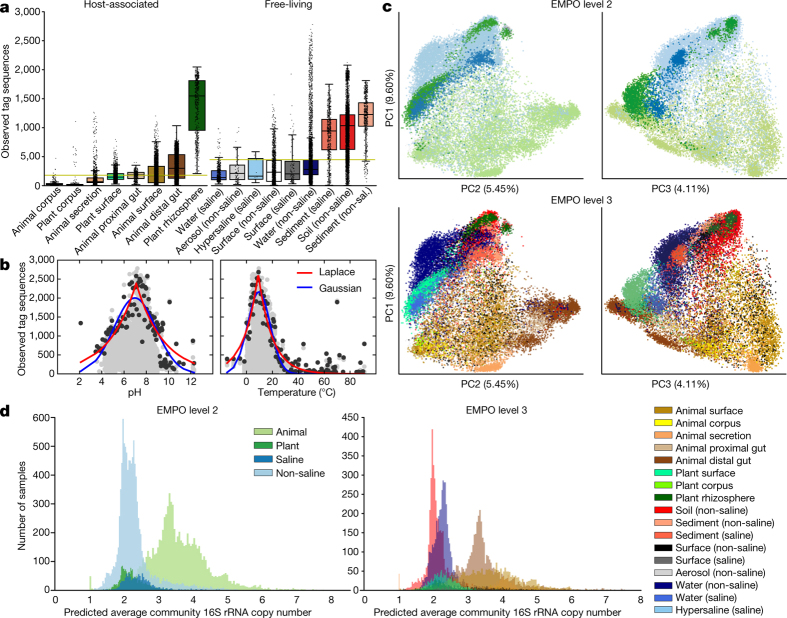
Alpha-diversity, beta-diversity, and predicted average 16S rRNA gene copy number. **a**, Within-community (alpha) diversity, measured as number of observed 90-bp tag sequences (richness), in *n* = 23,828 biologically independent samples as a function of environment (per-environment *n* shown in Fig. 1a), with boxplots showing median, interquartile range (IQR), and 1.5 × IQR (with outliers). Tag sequence counts were subsampled to 5,000 observations. Yellow line indicates the median number of observed tag sequences for all samples in that set of boxplots. Free-living communities of most types exhibited greater richness than host-associated communities. **b**, Tag sequence richness (as in **a**) versus pH and temperature in *n* = 3,986 (pH) and *n* = 6,976 (temperature) biologically independent samples. Black points are the 99th percentiles for richness across binned values of pH and temperature. Laplace (two-sided exponential) curves captured apparent upper bounds on microbial richness and their peaked distributions better than Gaussian curves. Greatest maximal richness occurred at values of pH and temperature that corresponded to modes of the Laplace curves. Maximum richness exponentially decreased away from these apparent optima. **c**, Between-community (beta) diversity among in *n* = 23,828 biologically independent samples: principal coordinates analysis (PCoA) of unweighted UniFrac distance, PC1 versus PC2 and PC1 versus PC3, coloured by EMPO levels 2 and 3. Clustering of samples could be explained largely by environment. **d**, 16S rRNA gene average copy number (ACN, abundance-weighted) of EMP communities in *n* = 23,228 biologically independent samples, coloured by environment. EMPO level 2 (left): animal-associated communities had a higher ACN distribution than plant-associated and free-living (both saline and non-saline) communities. Right: soil communities had the lowest ACN distribution, while animal gut and saliva communities had the highest ACN distribution. PowerPoint slide Source data

**Extended Data Figure 5:**
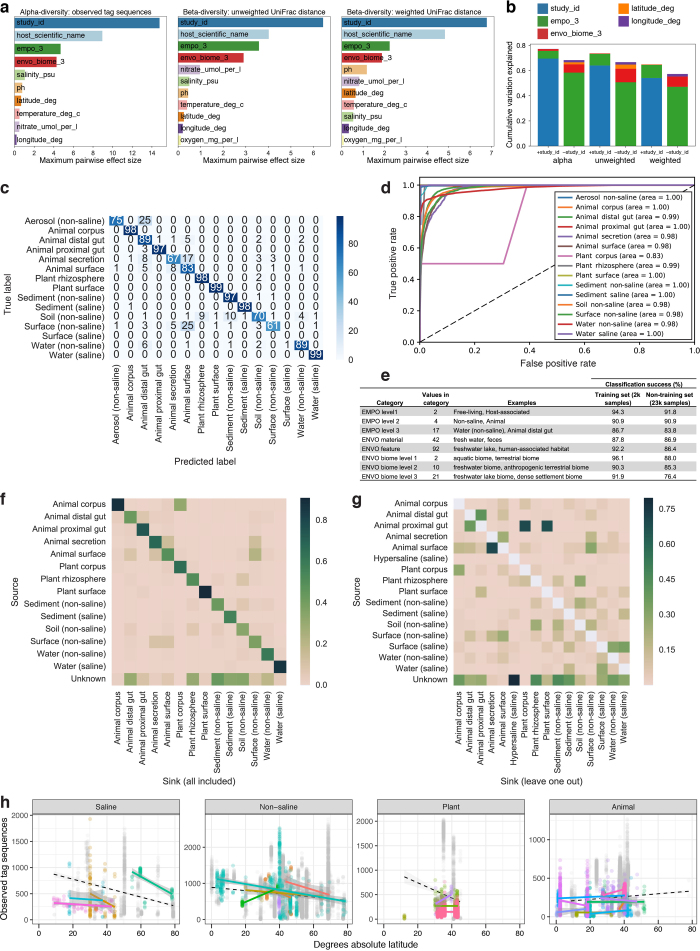
Environmental effect sizes, sample classification, and correlation patterns. **a**, Effect sizes of predictors on alpha- and beta-diversity. Maximum pairwise effect size (difference between means divided by standard deviation) between categories of each predictor plotted for observed tag sequences (alpha-diversity) and unweighted and weighted UniFrac distance (beta-diversity). Response variables (alpha- and beta-diversity) were derived from the QC-filtered subset of the 90-bp Deblur table containing *n* = 23,828 biologically independent samples. Numeric predictor variables were converted to quartiles (categorical predictors). Categories within each predictor had a minimum of 75 samples per category. **b**, Cumulative variation explained by the optimal model of stepwise redundancy analysis (RDA) of predictors: study ID, EMPO level 3, ENVO biome level 3, latitude, and longitude (predictors with values for less than half of samples, including host scientific name, were excluded). Environment (EMPO level 3) and biome (ENVO biome level 3) explained as much variation as study ID when study ID was excluded from the RDA. **c**, Confusion matrix for random forest classifier of samples to environment (EMPO level 3). The classifier was trained on the 2,000-sample subset, which was then tested on the remaining samples (QC-filtered samples minus 2,000-sample subset). Squares are coloured relative to 100 classification attempts for each true label. Overall success rate was 84%, with the most commonly misclassified sample environments being Surface (non-saline), Animal secretion, Soil (non-saline), and Aerosol (non-saline). **d**, Receiver operating characteristic (ROC) curve for classification of samples to environment (EMPO level 3). The AUC (area under curve) indicates the probability that the classifier will rank a randomly chosen sample of the given class higher than a randomly chosen sample of other classes. **e**, Classification success, using a random forest classifier, to EMPO levels 1–3, ENVO material, ENVO feature, and ENVO biome levels 1–3. **f**, Microbial source tracking: mean predicted proportion of tag sequences from each source environment (EMPO level 3) that occurs in each sink environment. The model was trained on a subset of samples (~20% of each environment), and tested to predict tag sequence source composition in all remaining samples. Aerosol (non-saline), Surface (saline), and Hypersaline samples were not included in this analysis because there were insufficient sample numbers. **g**, Microbial source tracking: which other environments a sample type most resembles. The model was trained on all source environments except one using a leave-one-out cross-validated model, and then used to classify each sample included in that group. Hence, the predicted classification proportion of environment *X* to environment *X* is zero. **h**, Correlation of microbial richness with latitude. Richness of 16S rRNA tag sequences per sample across EMPO level 2 environmental categories as a function of absolute latitude. Samples from studies that span at least 10° latitude are highlighted in colour, with linear fits displayed per-study as matching coloured lines. Samples from studies with narrower latitudinal origins are shown in grey. The global fit for all samples per category is indicated by a dashed black line.

We used a structured categorical variable of microbial environments, EMPO, to analyse diversity in the EMP catalogue in the context of lessons from previous investigations^1,2^. We observed environment-dependent patterns in the number of observed tag sequences (alpha-diversity), turnover and nestedness of taxa (beta-diversity), and predicted genome properties (ecological strategy). Derived from a more standardized methodology, our dataset confirms the previous finding^2^ that host association is a fundamental environmental factor that differentiates microbial communities (Fig. 2c, Extended Data Fig. 2d). We build on this pattern by showing that there is less richness in host-associated communities than in free-living communities (Fig. 2a), with the noted exception of plant rhizosphere samples, which resemble free-living soil communities in both richness (Fig. 2a) and composition (Fig. 2c). Our findings also confirm the major compositional distinction between saline and non-saline communities^1^ (Fig. 2c). The effect sizes of environmental factors on alpha- and beta-diversity generally showed large contributions of environment type and (for host-associated samples) host species to both types of diversity (Extended Data Fig. 5a, b).

The ability to identify sample provenance using only a microbial community profile has applications ranging from criminal forensics to mistaken sample identification; these applications will require large curated datasets, such as the EMP. Supervised machine learning demonstrated that samples could be distinguished as being animal-associated, plant-associated, saline free-living, or non-saline free-living with 91% accuracy based solely on community composition, and to fine-scale environment with 84% accuracy (Extended Data Fig. 5c–e). The most commonly misclassified samples were soil, non-saline surface and aerosol, and animal secretion. In many of these cases, misclassification can be attributed to the limitations of EMPO. As additional samples are classified, classification can be improved by iteratively and empirically redefining categories using machine learning. Conversely, with continuous factors, such as salinity, categorical definitions cannot perfectly capture intermediate values. High classification success to environment type was supported by source-tracking analyses (Extended Data Fig. 5f, g), with the exception of plant rhizosphere samples, owing to their similarity to soil samples.

Predicted average community copy number (ACN) of the 16S rRNA gene was another metric found to differentiate microbial communities in both host-associated and free-living communities (Fig. 2d). ACN can be predicted from 16S rRNA amplicon data^27^; this method has been used, for example, to link the taxonomic groups associated with copiotrophic and oligotrophic behaviours in soils to high and low rRNA gene copy numbers, respectively^28^. Approximately half the dataset centred on an ACN of 2.2 (free-living and plant-associated samples) and the other half on an ACN of 3.4 (animal-associated samples) (Fig. 2d). Greater per-genome rRNA operon copy number has been found to be associated with rapid maximum growth rates^29^, which may provide a selective advantage when resources are abundant, such as in animal hosts. While ACN is an estimate rather than a measurement of average rRNA copy number and is subject to potential biases in the underlying reference database, the distributions we observed are consistent with 16S rRNA copy number reflecting differences in ecological strategies among environments.

## A resource for theoretical ecology

The coordinated accumulation of data across studies allows investigations of patterns within (alpha-diversity) and among (beta-diversity) microbial communities at scales that vastly exceed what could be measured in any individual study. Patterns of alpha-diversity in meta-analyses have revealed global trends that have been key to the development of major ideas in macroecological theory, but fundamental patterns have been more difficult to discern in microbial ecology. For example, a nearly ubiquitous tendency towards greater diversity in the tropics is evident in macroecology^30^, but there is substantial variation among studies examining latitudinal trends of microbial diversity^31,32,33^. The large EMP dataset analysed here reveals a weak but significant trend towards increasing diversity at lower latitudes in non-host-associated environments (Extended Data Fig. 5h). An effect of latitude was apparent both within and across studies, consistent with global trends in latitudinal microbial diversity being an emergent function of locally selective environmental heterogeneity^34^. However, substantial study-to-study variation in richness highlights the caveats inherent in meta-analysis; more coordination of sample collections from similar environments across larger gradients is necessary to better address this question.

The EMP has the potential to link global patterns of microbial diversity with physicochemical parameters—if appropriate metadata are provided by researchers. Microbial community richness has been found to correlate with environmental factors, including pH and temperature^3,33,35,36^. For example, richness has been shown to increase up to neutral pH^36^ and often to decrease above neutral pH^3,35^ in soil communities. Richness has been shown to increase with temperature up to a limit and then to decrease beyond that limit in seawater (maximum at about 19 °C)^33^ and to increase with temperature in soil (up to at least around 26 °C)^36^. However, general relationships of richness to temperature and pH remain unresolved^37^. Here, across samples from non-host-associated environments where pH or temperature were measured (mostly freshwater and soil environments), richness was greatest near neutral pH (around 7) and relatively cool temperatures (about 10 °C) (Fig. 2b). We observed apparent upper bounds on richness with both temperature and pH that were best fit by two-sided exponential (Laplace) curves. Thus, the present dataset suggests that maximum microbial richness occurs within a relatively narrow range of intermediate pH and temperature values. These patterns, while robust in the context of the EMP dataset, necessarily reflect only the subset of sample types for which variables were measured (Supplementary Table 2); they should therefore be interpreted with caution. Understanding universal relationships between richness and environmental factors will require information from more studies with detailed and carefully collected physicochemical metadata.

**Figure 3:**
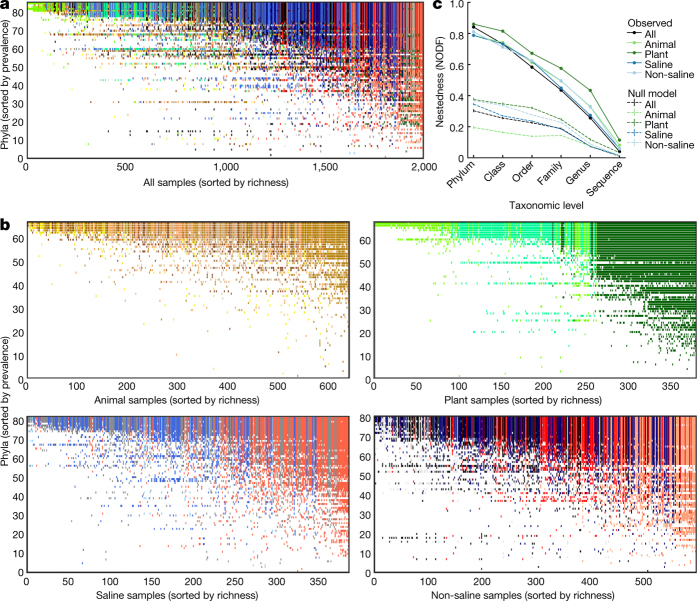
Nestedness of community composition. **a**, Presence–absence of phyla across samples, with phyla (rows) sorted by prevalence and samples (columns) sorted by richness. Shown is a subset of the EMP consisting of *n* = 2,000 biologically independent samples with even representation across environments and studies. With increasing sample richness (left to right), phyla tended to be gained but not lost (*P* < 0.0001 versus null model; NODF (nestedness measure based on overlap and decreasing fills) statistic and 95% confidence interval = 0.841 ± 0.018). **b**, As in **a** but separated into non-saline, saline, animal, and plant environments (*P* < 0.0001, respective NODF = 0.811 ± 0.013, 0.787 ± 0.015, 0.788 ± 0.018 and 0.860 ± 0.021). **c**, Nestedness as a function of taxonomic level, from phylum to tag sequence, across all samples and within environment types. Also shown are median null model NODF scores (± s.d.). NODF measures the average fraction of taxa from less diverse communities that occur in more diverse communities. All environments at all taxonomic levels were more nested than expected randomly, with nestedness higher at higher taxonomic levels (for example, phyla). PowerPoint slide Source data

**Extended Data Figure 6:**
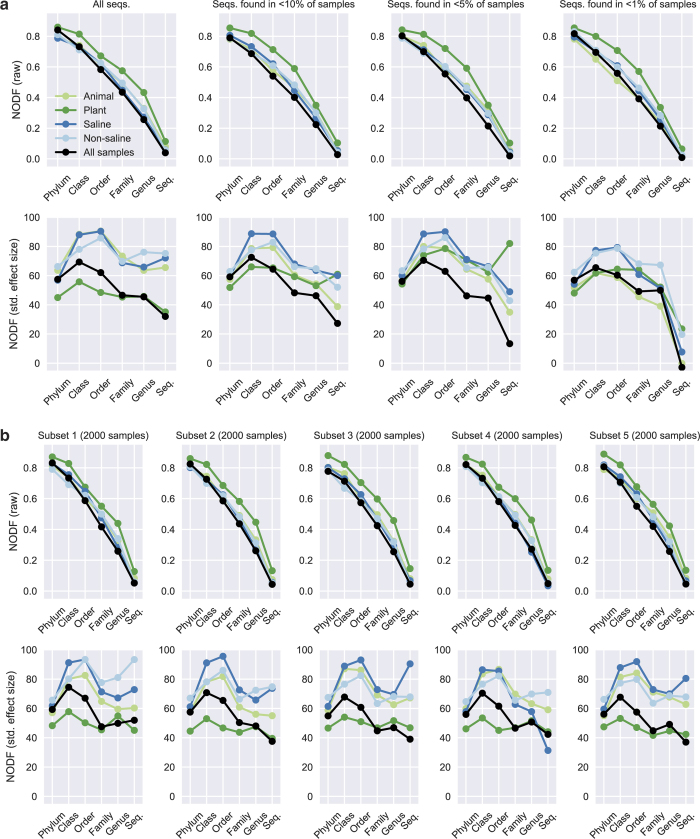
NODF scores of nestedness across samples by taxonomic level. The NODF statistic represents the mean, across pairs of samples, of the fraction of taxa occurring in less diverse samples that also occur in more diverse samples. A raw NODF of 0.5 would mean that for any pair of samples, on average 50% of the taxa in the less diverse sample would occur in the more diverse sample. **a**, NODF (raw) and NODF standardized effect size in the 2,000-sample subset by taxonomic level. Results are shown first for all tag sequences and then for tag sequences found in <10%, < 5%, and <1% of samples. By removing the most prevalent tag sequences before analysis (and rarefying only after this step), it was possible to rule out artefacts associated with potential contamination. NODF (raw) is highest at the phylum level and decreases at finer taxonomic levels, and this trend is observed even when the most prevalent tag sequences are removed (removing those occurring in ≥ 10%, ≥ 5%, or ≥ 1% of samples). The decreasing trend is likely to be partially due to finer taxonomic groups having lower prevalence (and lower matrix fill, among other factors) than coarser taxonomic groups, as standardized effect sizes of the NODF statistic are essentially constant across taxonomic levels. **b**, When five alternate 2,000-sample subsets are randomly drawn (with replacement) from the full (QC-filtered) EMP dataset, the trends in NODF (raw) and NODF standardized effect size remain largely unchanged.

Beyond measured physical covariates, the breadth of environments in the EMP catalogue allows a detailed exploration of how microbial diversity is distributed across environments. Diversity among communities (beta-diversity) is driven by turnover (replacement of taxa) and nestedness (gain or loss of taxa resulting in differences in richness)^38^. If turnover dominates, then disparate communities will harbour unique taxa. If nestedness dominates, then communities with fewer taxa will be subsets of communities with more taxa. We tested for nestedness using a 2,000-sample subset with even representation across environments and studies. Given the contrasting environments and geographic separation among the many studies in the EMP, we expected different environments to contain unique sets of taxa and to show little nestedness. However, we found that communities across environments were significantly nested (Fig. 3a, b; *P* < 0.05) in comparison to null models (Fig. 3c), accounting for the observed patterns of richness. At coarse taxonomic levels, an average of 84% of phyla, 73% of classes, and 58% of orders that occurred in less diverse samples also occurred in more diverse samples. Nestedness was observed even when the most prevalent taxa were removed and was robust across randomly chosen subsets of samples (Extended Data Fig. 6). These patterns could have resulted from several mechanisms, including ordered extinctions^39^ and the filtering of complex communities over time^40^, differential dispersal abilities^41^ and cascading source–sink colonization processes that assemble nested subsets from more complex communities, or by the tendency of larger habitat patches to support more rare taxa with lower prevalence^42^. Notably, finer taxonomic groupings showed less nestedness (Fig. 3c), indicating that the processes that underlie nested patterns of turnover are likely to reflect conserved aspects of microbial biology, and not to result from the interplay of diversification and dispersal on short timescales.

These global ecological patterns offer a glimpse of what is possible with coordinated and cumulative sampling—in addition to the specific questions addressed by individual studies, context is built and easily queried across studies. They also necessarily highlight the inherent limitations to decentralized studies, especially regarding the collection of comparable environmental data. Future studies will be able to use the current EMP data as a starting point for more explicit tests of broad ecological principles, both to identify gaps in current knowledge and to more confidently plan large directed studies with sufficient power to fill them.

## A more precise and scalable catalogue

**Figure 4:**
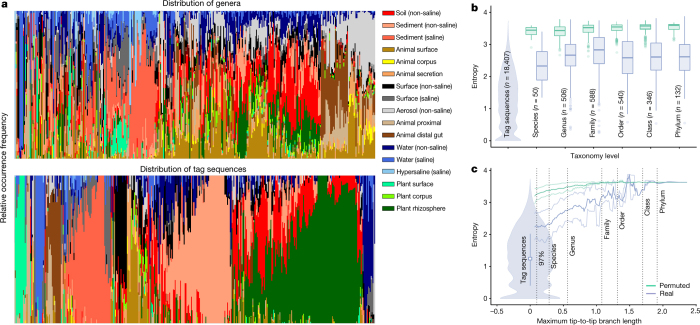
Specificity of sequences and higher taxonomic groups for environment. **a**, Environment distribution in all genera and 400 randomly chosen tag sequences, drawn from *n* = 2,000 biologically independent samples with even representation across environments (EMPO level 3) and studies. Each bar depicts the distribution of environments for a taxon (not relative abundance of taxa): bars composed mostly of one colour (environment) are specific for that environment, as seen with tag sequences; bars composed of many colours are more cosmopolitan, as seen with genera. Tag sequences were more specific for environment than were genera and higher taxonomic levels. **b**, Shannon entropy within each taxonomic group (minimum 20 tag sequences per group) and for the same set of samples with permuted taxonomy labels. Box plots show median, IQR, and 1.5 × IQR (with outliers) for each taxonomic level. A violin plot shows the entropy of tag sequences (minimum 10 samples per tag sequence). Specificity for environment occurred predominantly below the genus level. **c**, Shannon entropy within phylogenetic subtrees of tag sequences (minimum 20 tips per subtree) defined by maximal tip-to-tip branch length (substitutions per site) and for the same samples with permuted phylogenetic tree tips. Mean and 20th/80th percentile for a sliding window average of branch length is shown. Violin plot for tag sequences as in **b**. Dotted lines show average tip-to-tip branch length corresponding to 97% sequence identity and taxonomic levels displayed in **b**. The greatest decrease in entropy was between the lowest branch length subtree tested and tag sequences. PowerPoint slide Source data

An advantage of using exact sequences is that they enable us to observe and analyse microbial distribution patterns at finer resolution than is possible with traditional OTUs. As an example, we applied a Shannon entropy analysis to tag sequences and higher taxonomic groups to measure biases in the distribution of taxa. Taxa that are equally likely to be found in any environment will have high entropy and low specificity, whereas taxa found only in a single environment will have low entropy and high specificity (note that we use ‘specificity’ solely to denote distributional patterns, not to imply adaptation or causality). Tag sequences exhibited high specificity for environment, with distributions skewed towards one or a few environments (low Shannon entropy); by contrast, higher taxonomic levels tended to be more evenly distributed across environments (high Shannon entropy, low specificity) (Fig. 4a). Entropy distributions across all tag sequences at each taxonomic level show that this pattern is general (Fig. 4b). Seeking a more precise measure of the divergence at which a taxon is specific for environments, we next investigated how entropy changes as a function of phylogenetic distance. We calculated entropy for each node of the phylogeny and visualized it as a function of maximum tip-to-tip branch length (Fig. 4c). While entropy decreased gradually at finer phylogenetic resolution, it dropped sharply at the tips of the tree. We conclude that environment specificity is best captured by individual 16S rRNA sequences, below the typical threshold defining microbial species (97% identity of the 16S rRNA gene).

**Extended Data Figure 7:**
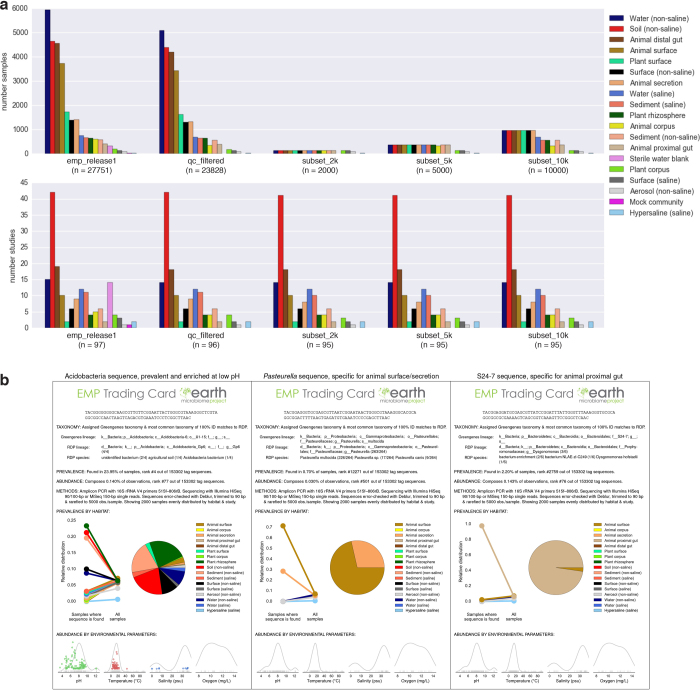
Subsets and EMP trading cards. **a**, Subsets of the EMP dataset with even distribution across samples and studies. Shown are all EMP samples included in this manuscript (release 1), the QC-filtered subset, and subsets of 10,000, 5,000, and 2,000 samples. The latter three contain progressively more even representation across environments and studies, providing a more representative view of the Earth microbiome and a more lightweight dataset. Top, histograms of samples per environment (EMPO level 3) for each subset. Bottom, histograms of studies per environment (EMPO level 3) for each subset. **b**, EMP trading cards: distribution of 16S rRNA tag sequences across the EMP. Trading cards highlight the power of the EMP dataset to help define niche ranges of individual microbial sequence types across the planet’s microbial communities. Cards show distribution of 16S rRNA tag sequences in a 2,000-sample subset of the EMP (rarefied to 5,000 observations per sample) having even distribution by environment (EMPO level 3) and study. Taxonomy is from Greengenes 13.8 and Ribosomal Database Project (RDP), with the fraction of exact RDP matches by lineage and species name shown in parentheses. The pie chart and point plot show the relative distribution of environments in which the tag sequence is found (left points) versus the environment distribution of all 2,000 samples (right points). The coloured scatter plots indicate tag sequence relative abundance (normalized to the shared *y* axis) as a function of metadata values (no points shown indicates that metadata were not provided for that category). For comparison, grey curves with rug plots indicate kernel density estimates of metadata values across all samples in the set of 2,000 (not just samples where the tag sequence was found). Three examples are shown. Left, a prevalent sequence enriched in soil and plant rhizosphere is from the class Acidobacteria, aptly named as this sequence is found at highest relative abundance in low-pH samples. Middle, the sequence most specific for animal surface (also enriched in animal secretion) is annotated as *Pasteurella multocida*, a common cause of zoonotic infections following bites or scratches by domestic animals, such as cats and dogs^83^. Right, the sequence most specific for animal proximal gut belongs to S24-7, a family highly localized to the gastrointestinal tracts of homeothermic animals and predominantly found in herbivores and omnivores, but not in carnivores^84^.

The EMP dataset provides the ability to track individual sequences across the Earth’s microbial communities. Using a representative subset of the EMP (Extended Data Fig. 7a), we produced a table of sequence counts and distributions, including among environments (EMPO) and along environmental gradients (pH, temperature, salinity, and oxygen). From this we generated ‘EMP Trading Cards’, which promote exploration of the dataset and here highlight the distribution patterns of three prevalent or environment-correlated tag sequences (Extended Data Fig. 7b, Supplementary Table 3). The entire EMP catalogue can be queried using the Redbiom software, with command-line (https://github.com/biocore/redbiom) and web-based (http://qiita.microbio.me) interfaces to find samples based on sequences, taxa, or sample metadata, and to export selected sample data and metadata (instructions at https://github.com/biocore/emp). User data generated from the EMP protocols can be readily incorporated into this framework: because Deblur operates independently on each sample^21^, additional tag sequences can be added to this dataset from new studies without reprocessing existing samples. Future combinations of datasets targeting the same genomic region but sequenced using different methods may be admissible but would require considerations to account for methodological biases.

The growing EMP catalogue is expected to have applications for research and industry, with tag sequences used as environmental indicators and representing targets for cultivation, genome sequencing, and laboratory study. In addition, these tools and approaches, although developed for bacteria and archaea, could be expanded to all domains of life^43^. To achieve greater utility for the EMP and similar projects, we must continually improve metadata collection and curation, ontologies, support for multi-omics data, and access to computational resources.

## Conclusions and future directions

Here we have used crowdsourced sample collection and standardized microbiome sequencing and metadata curation to perform a global meta-analysis of bacterial and archaeal communities. Using exact sequences in place of OTUs and a learned structure of microbial environments, we have shown that agglomerative sampling can reveal basic biogeographic patterns of microbial ecology, with resolution and scope rivaling data compilations currently available for ‘macrobial’ ecology^44,45^. Our results point to key organizing principles of microbial communities, with less-rich communities nested within richer communities at higher taxonomic levels, and environment specificity becoming much more evident at the level of individual 16S rRNA sequences.

The EMP framework and global synthesis presented here represent value added to the scientific community beyond the substantial contributions of the constituent studies (Supplementary Table 1). However, as with any meta-analysis in which data are gathered primarily in service of separate questions rather than a single theme^46^, conclusions should be viewed with caution and form starting points for future hypothesis-directed investigations. There is a need to span gradients of geography (for example, latitude and elevation) and chemistry (for example, temperature, pH, and salinity) more evenly—assisted by tools for more comprehensive collection and curation of metadata—and to track environments over time. In addition, biotic factors (for example, animals, fungi, plants, viruses, and eukaryotic microbes) not measured in this study have important roles in determining community structure^4,5,6^. The scalable framework introduced here can be expanded to address these needs: new studies to fill gaps in physicochemical space, amplicon data for microbial eukaryotes and viruses, and whole-genome and whole-metabolome profiling. At a time when both academic and governmental agencies increasingly recognize the value of communal biodiversity monitoring efforts^47,48^, the EMP provides one example of a logistically feasible standardization framework to maximize interoperability across diverse and independent studies, in particular using stable identifiers (exact sequences) to enable enduring utility of environmental sequence data. Given current global sequencing efforts, the use of coordinated protocols and submission to this and other public databases should allow rapid accumulation of new data, providing an ever more diverse reference catalogue of microbes and microbiomes on Earth.

## Methods

### Study design

This effort was possible because of a unified standard workflow that leveraged existing sample and data reporting standards to allow biomass and metadata collection across diverse environments on Earth. After sample collection, all samples were processed following the same protocols. A standard DNA extraction protocol (http://www.earthmicrobiome.org/protocols-and-standards/dna-extraction-protocol) was implemented to ensure that trends observed were due either to the biological system or to biases in extraction potential for organisms from different environmental matrices, and not due to inherent biases in the extraction protocol. To avoid known issues that arise when multiple amplicon strategies are combined^49^, we also standardized PCR primers, amplification strategy, and sequencing^50^. More recent studies not included in this meta-analysis adopted additional primer modifications to allow recovery of key taxa in marine and soil samples^17,18,19^. Data reporting standards, including the MIxS (minimal information about any sequence) metadata standard developed by the Genomic Standards Consortium^10^ and the Environment Ontology (ENVO)^11,51^, enabled interoperability, data analysis, and interpretation between samples from disparate environments, collected using many different techniques through unconnected programs of investigation.

To transfer our knowledge of microbial environments to the broader community, we engaged with the developers of ENVO to ensure that the basic, salient features of microbial environments (host-associated or free-living, and respectively within those, animal- or plant-associated, and saline or non-saline materials) were represented either in this ontology or in those with which it interoperates. For ease of application, we gathered these contributions into an application ontology, the EMP Ontology (EMPO) (Fig. 1a). The EMP community will continue to work with ontology engineers to shape ENVO and other ontologies around the EMPO application ontology. EMPO will be maintained as a logical subset of ENVO and integrated into the ENVO release cycle to maximize interoperation.

Metadata curation was automated using Pandas (http://pandas.pydata.org). The size of the dataset also required extensive software development to support analysis at this scale, leading to tools including the data and analysis portal Qiita (http://qiita.microbio.me), the BIOM format^52^, new ‘OTU picking’ methods Deblur^21^ and a subsampled open-reference procedure^53^, a scalability improvement of Fast UniFrac phylogenetic inference software^54^, speed improvements to sequence-insertion tree method SEPP^55^, and speed and feature improvements to Emperor ordination visualization software^56^ (http://biocore.github.io/emperor).

### Sample collection

The global community of microbial ecologists was invited to submit samples for microbiome analysis, and samples were accepted for DNA extraction and sequencing provided that scientific justification and high-quality sample metadata were provided before sample submission. Standardized sampling procedures for each sample type were used by contributing investigators. Samples were collected fresh and, where possible, immediately frozen in liquid nitrogen and stored at –80 °C. Detailed sampling protocols are described in publications of the individual studies (Supplementary Table 1). Bulk samples (for example, soil, sediment, faeces) and fractionated bulk samples (for example, sponge coral surface tissue, centrifuged turbid water) were taken using microcentrifuge tubes. Swabs (BD SWUBE dual cotton swabs or similar) were used for biofilm or surface samples. Filters (Sterivex cartridges, 0.2 μm, Millipore) were used for water samples. Samples were sent to laboratories in the United States for DNA extraction and sequencing: water samples to Argonne National Laboratory, soil samples to Lawrence Berkeley National Laboratory (pre-2014) or Pacific Northwest National Laboratory (2014 onward), and faecal and other samples to the University of Colorado Boulder (pre-2015) or the University of California San Diego (2015 onward).

### Metadata curation and EMP ontology

Metadata were collected in compliance with MIMARKS^10^, EBI (https://www.ebi.ac.uk/ena), and Qiita (http://qiita.microbio.me) standards, as described in the EMP Metadata Guide (http://www.earthmicrobiome.org/protocols-and-standards/metadata-guide). QIIME mapping files (metadata) were downloaded from Qiita, merged, and refined using Python with Pandas, generating quality-controlled mapping files. Mapping file columns are described in Supplementary Table 2. Mapping files for the full EMP dataset and subsets (see below) are available at ftp://ftp.microbio.me/emp/release1/mapping_files/. The EMP Ontology (EMPO) for microbial environments was devised to facilitate the present analysis while preserving interoperability. Coordinated by the ENVO team, annotations from ENVO^11,51^, UBERON (metazoan anatomy)^57^, PO (plant ontology)^58^, FAO (fungal anatomy ontology, http://purl.obolibrary.org/obo/fao.owl), and OMP (ontology of microbial phenotypes)^59^ were mapped to our EMPO levels 2 and 3 (empo_2 and empo_3). Additionally, the free-living or host-associated lifestyles were captured in level 1 categories (empo_1). Descriptions of empo_3 categories are provided at http://www.earthmicrobiome.org/protocols-and-standards/empo. The W3C Web Ontology Language (OWL) document is available at http://purl.obolibrary.org/obo/envo/subsets/envoEmpo.owl. Map data were derived from the open-source project MatPlotLib package Basemap, which distributes map data from Generic Mapping Tools data (http://gmt.soest.hawaii.edu) released under the GNU Lesser General Public License v3.

### DNA extraction, amplicon PCR, sequencing, and sequence pre-processing

DNA extraction and 16S rRNA amplicon sequencing was done using EMP standard protocols (http://www.earthmicrobiome.org/protocols-and-standards/16s)^14^. In brief, DNA was extracted using the MO BIO PowerSoil DNA extraction kit (Carlsbad, CA), chosen because of its versatility with diverse sample types (rather than high yields with any given sample type). Amplicon PCR was performed on the V4 region of the 16S rRNA gene using the primer pair 515f–806r^50^ with Golay error-correcting barcodes on the reverse primer. Although any primer-based method necessarily under-samples diversity, a recent analysis of 16S rRNA genes captured in shotgun metagenomic sequences indicates that this primer pair is among the best available for sampling both bacteria and non-eukaryotic archaea^15^. Amplicons were barcoded and pooled in equal concentrations for sequencing. The amplicon pool was purified with the MoBio UltraClean PCR Clean-up kit and sequenced on the Illumina HiSeq or MiSeq sequencing platform; the same sequencing primers were used with both platforms, and previous work has shown that conclusions drawn from 16S rRNA amplicon data are not dependent on which of these sequencing platforms is used^50^. Sequence data were demultiplexed and minimally quality filtered using the QIIME 1.9.1 script split_libraries_fastq.py^60^ with Phred quality threshold of 3 and default parameters to generate per-study FASTA sequence files.

### Tag sequence and OTU picking and subsets

Sequence data were error-filtered and trimmed to the length of the shortest sequencing run (90 bp) using the Deblur software^21^; the resulting 90-bp Deblur BIOM table was used for all analyses unless otherwise noted. Deblur tables trimmed to 100 bp and 150 bp were also generated and provided, which contain greater sequence resolution but fewer samples. Deblur observation tables were filtered to keep only tag sequences with at least 25 reads total over all samples. For comparison to existing OTU tables, traditional closed-reference OTU picking was done against 16S rRNA databases Greengenes 13.8^25^ and SILVA 123^26^ using SortMeRNA^61^, and subsampled open-reference OTU picking^53^ was done against Greengenes 13.8. These unfiltered tables and the filtered and subset tables described below are available at ftp://ftp.microbio.me/emp/release1/otu_tables.

A total of 97 studies and 27,742 samples are included in the present study and in the unfiltered BIOM tables. The QC-filtered subset used in core diversity analyses (Fig. 2) contains 96 studies and 23,828 samples, and it was subset further for some analyses. In the provided BIOM tables (ftp://ftp.microbio.me/emp/release1/otu_tables/ and https://zenodo.org/record/890000), the ‘release1’ set contains all samples in the 97 studies that have at least one sequence per sample; this set includes controls (blanks and mock communities). The ‘qc_filtered’ set, from which the subsets are drawn, has samples with ≥ 1,000 observations in each of four observation tables: closed-reference Greengenes, closed-reference SILVA, open-reference Greengenes, and Deblur 90-bp; controls (empo_1 == ‘Control’) are excluded. Subsets were then generated which give equal (as possible) representation across environments (EMPO level 3) and across studies within those environments. The subsets contain 10,000, 5,000, and 2,000 samples (nested subsets). In each subset the samples must have ≥ 5,000 observations in the Deblur 90-bp observation table and ≥ 10,000 observations in each of the closed-reference Greengenes, closed-reference SILVA, and open-reference Greengenes observation tables. Note that Deblur removes approximately one-third to one-half of sequences owing to suspected errors, which is consistent with a sequence length of ~90–150 bp and an average error rate of 0.006 per position^62^.

### Comparison against reference databases

To compare the unique sequence diversity in this study to that in existing databases, sequences from the complete Deblur 90-bp observation table were compared to the set of unique full-length sequences from Greengenes 13.8 and the non-eukaryotic fraction of Silva 128 databases using the open-source sequence search tool VSEARCH^63^ in global alignment search mode, requiring 100% similarity across the query sequence and allowing multiple 100% reference matches.

### Prevalence as a function of sequencing depth

The QC-filtered Deblur 90-bp observation table was additionally filtered to samples that had at least 50,000 sequences (observations). We chose to focus on four environment types (EMPO level 3) where there were many hundreds of samples with more than 50,000 sequences: soil (*n* = 2,279), saltwater (*n* = 478), freshwater (*n* = 1,508), and animal distal gut (*n* = 695) environments. For each environment, the observation tables were randomly subsampled to 50, 500, 5,000, and 50,000 sequences per sample. The prevalence of each tag sequence was determined as the number of non-zero occurrences across samples divided by the total number of samples. We then plotted a histogram of tag sequence prevalence at each sampling depth. In order to control for potential study bias, we ran the same analysis on a subset of the observation tables where 30 samples were randomly sampled from each study (studies with fewer than 30 samples with > 50,000 sequences were discarded). To investigate how mean tag sequence prevalence changes with increasing sequencing depth across environments, we calculated the average mean tag sequence prevalence across three replicate rarefactions. We plotted the average and standard deviation in mean prevalence across replicate subsamples over a subsampling gradient (that is, 50, 100, 500, 1,000, 5,000, 10,000 and 50,000 sequences per sample).

### Greengenes insertion tree

Deblur tag sequences were inserted into the Greengenes reference tree using SEPP^55^, which uses a divide-and-conquer technique to enable phylogenetic placement on very large reference trees. The SEPP method uses HMMER^64^ internally for aligning each Deblurred sequence to a reference Greengenes alignment (gg_13_5_ssu_align_99_pfiltered.fasta) with 99% threshold for clustering (resulting in 203,452 tag sequences) and dividing the reference alignment to subsets with a thousand sequences each. It then uses pplacer^65^ to insert the sequences into the reference Greengenes tree (99_otus.tree), dividing it into subsets of size 5,000. The branch lengths on the Greengenes tree were recomputed using RAxML^66^ under the GTRCAT model before the placement. The pipeline used, including the reference trees and alignments can be found at ftp://ftp.microbio.me/emp/release1/otu_info/greengenes_sepp_pipeline, and the bash script is available at https://github.com/biocore/emp/blob/master/code/03-otu-picking-trees/deblur/run_sepp.sh.

### Fast UniFrac

Unweighted and weighted UniFrac were computed using the Cythonized^67^ implementation of Fast UniFrac^54^ in scikit-bio^68^. Fast UniFrac by itself was not scalable for the EMP dataset owing to an intermediary data structure required by the algorithm, which scales in space by *O*(*NM*), where *N* is the number of nodes in the phylogeny and *M* is the number of samples. A workaround was designed and implemented in scikit-bio (skbio.diversity.block_beta_diversity) which computes partial distance matrices as opposed to all samples pairwise, enabling large reductions within the intermediary data structure by shrinking *M* and, in tandem, shrinking *N* to only the relevant nodes of the phylogeny. This decomposition also allows a classic map-reduce parallel approach with low per-process space requirements. Further space and time reductions were obtained through the implementation and use of a balanced-parentheses tree representation^69^ (https://pypi.python.org/pypi/iow).

### Core diversity analyses: alpha- and beta-diversity

Alpha-rarefaction was computed using single_rarefaction.py in QIIME 1.9.1^60^ using as input the Deblur 90-bp BIOM table and rarefaction depths of 1,000, 5,000, 10,000, 30,000, and 100,000. Alpha-diversity was computed using scikit-bio 0.5.0 with the input Deblur 90-bp BIOM table rarefied to 5,000 observations per sample, and alpha-diversity indices were observed_otus (number of unique tag sequences), shannon (Shannon diversity index^70^), chao1 (Chao 1 index^71^), and faith_pd (Faith’s phylogenetic diversity^72^, using the Greengenes insertion tree). Fast UniFrac^54^ was run on the Deblur 90-bp table using the aforementioned approach and the corresponding insertion tree. Principal coordinates were computed using QIIME 1.9.1.

### Effect size calculations of alpha- and beta-diversity

A version of the mapping file (metadata) was compiled containing the predictors to be tested: study_id, host_scientific_name (a proxy for host taxonomy), latitude_deg, longitude_deg, envo_biome_3 (a proxy for biome or environment), empo_3 (a proxy for sample type or environment generally), temperature_deg_c, ph, salinity_psu, and nitrate_umol_per_l (a proxy for nutrient levels generally). Predictors chosen were those expected to be less redundant with other predictors not chosen, with the exception that there was substantial overlap between study ID and many of the other predictors—because independent studies typically focused on limited sample types from constrained geographic ranges, it is expected that study ID serves as a proxy for a wide range of other measured and unmeasured environmental variables (see Extended Data Fig. 5b). Categories for each predictor were chosen as follows: numerical data were first converted to categories using quartiles; then each category was required to be found in at least 0.3% of all samples (corresponding to 75 samples); categories that were less common than this were ignored. Note that some predictors in our data have complex nonlinear relationships that multivariate statistical analyses using quartiles may miss, such as the unimodal upper-constraint-based richness relationships of temperature and pH. We then tested the effect size of each predictor versus the number of observed tag sequences (alpha-diversity) and weighted and unweighted UniFrac distances (beta-diversity). Effect size was calculated using a Python implementation of the mixed-directional false discovery rate (mdFDR)^73,74^. mdFDR reduces the false discovery rates by penalizing the multiple pairwise comparisons within each metadata category and the multiple metadata category comparisons. mdFDR has four steps. First, it performs a pairwise comparison (Mann–Whitney *U* for alpha-diversity, and PERMANOVA for beta-diversity) of each group within each category. Second, for each category we calculate a pooled *P* value based on the *P* values of all pairwise comparisons for any given category. Third, we apply the Benjamini–Hochberg procedure to the pooled *P* values and remove non-significant metadata categories. Finally, we estimate the effect size of those categories found to be significant in step 3 and that have a pairwise comparison *P* value greater than (*R*/*m* × *q*_*i*_) × *α*, where *R* is the number of categories that were found significant, *m* is the number of categories that are being compared (the original number of categories in the input mapping file), *q*_*i*_ is the number of pairwise comparisons in each given category, and *α* is the control level for FDR. The effect size for a given metadata column is calculated as the difference of means of each pairwise comparison divided by pooled standard deviation. To further assess the combined effect size of predictors with non-redundant explanatory power on alpha- and beta-diversity, the non-redundant predictors were selected by forward stepwise redundancy analysis with the R package vegan^75^ ordiR2step function. This analysis provides an estimate of the relative contribution of each non-redundant predictor to the combined effect size and their independent fraction to the community variation.

### Average community 16S rRNA gene copy number

The closed-reference observation table (Greengenes 13.8) was run through the PICRUSt 1.1.0 command normalize_by_copy_number.py script^76^, which divides the abundance of each OTU by its inferred 16S rRNA gene copy number (that is, copy number is inferred from the closest genome representative for a Greengenes 16S rRNA gene reference sequence). Samples with more than 10,000 sequence reads were summed (that is, OTU abundances were summed within each sample) in both the copy-number normalized and original observation tables. The weighted average community 16S rRNA gene copy number (ACN) for each sample was calculated as the raw sample sum divided by the normalized sample sum.

### Covariation of richness with latitude, pH, and temperature

Measurements of alpha-diversity were compared to absolute latitude using a linear mixed-effects model incorporating study ID as a random variable and the interaction of environment and latitude as fixed effects; this was performed on a dataset filtered to include only studies comprising samples that spanned at least 10° of absolute latitude. Correlation of richness with pH and temperature were fitted with a Laplace distribution. The Laplace distribution is a continuous probability distribution that simultaneously captures exponential increase and exponential decrease around a modal value (*μ*). This distribution is also referred to as the double exponential or two-sided exponential because it represents two symmetrical exponential distributions back-to-back. The Laplace is particularly useful for testing the biological hypothesis that a system is under strong selection to take a particular value (*μ*) and that small deviations from *μ* produce an exponential decrease, for example, in diversity. We tested this hypothesis with regards to how tag sequence richness (*S*) relates to pH and temperature. We used the upper 99th percentile of tag sequence richness across narrow ranges of pH (100 bins) and temperature (120 bins), meaning that our question pertained to the relationship of maximum tag sequence richness (*S*_max_) to pH and temperature. We compared our expectations of exponential decrease in maximum *S* against the fit to a Gaussian curve, which can also predict a steep symmetrical decrease with small deviations from *μ*.

### Random forest classification of samples

Random forest classification models were trained on the 2,000-sample subset of the Deblur 90-bp observation table to test classification success of samples into the environmental categories from which they came. The R packages caret^77^ and randomForest^78^ were used. Five repeats of tenfold cross-validation were used to evaluate the classification accuracy. Confusion matrices were computed to measure the agreement between prediction and true observation. The models were then used to classify the other remaining samples in the full QC-filtered subset.

### SourceTracker analyses

SourceTracker^79^ uses a Bayesian classification model together with Gibbs sampling to predict the proportion of tag sequences from a given set of source environments that contribute to sink environments. We applied SourceTracker 2.0.1 (http://github.com/biota/sourcetracker2) to define the degree to which tag sequences are shared among environmental samples, using the 2,000-sample subset of the Deblur 90-bp observation table (~20% of each sample type) as source samples to train the model, and the remainder as sink samples to test the model. Additionally, we used leave-one-out cross-validation to predict the sample type of each source sample when that sample type is excluded from the model, in order to evaluate the homogeneity of source samples and independence of each source type. Source and sink samples were rarefied to 1,000 sequences per sample before feature selection and testing.

### Nestedness of taxonomic composition

Nestedness captures the degree to which elements of a large set are contained within progressively smaller sets. We used the NODF statistic^80^ to quantify nestedness of the sample-by-taxa matrix. The rows of this matrix correspond to specific taxa grouped at particular taxonomic levels (for example, phylum, class, etc.), while the columns correspond to particular samples. After sorting the matrix from greatest-to-least according to row and column sums, we quantified two aspects of the NODF statistic. The first is a ‘row’ version of NODF that quantifies the degree to which ranges of less prevalent taxa are subsets of the ranges of more prevalent taxa. The second is a ‘column’ version of NODF that quantifies the degree to which less diverse communities are subsets of more diverse communities. We employed two null models to better interpret the observed values of the NODF statistic. The first is based on a random shuffling of occurrences within each row, holding row sums constant (fixed rows, equiprobable columns), while the second is based on a random shuffling of occurrences within each column, holding column sums constant (equiprobable rows, fixed columns)^81^. The results from both of these null models were qualitatively consistent, so we only report findings using the equiprobable rows, fixed columns model, as it is more consistent with rarefaction of the observation tables. We considered null models at each taxonomic level (phylum, class, order, family, genus), and for all of the samples and each subset of the samples at EMPO level 2. To compute standardized effect scores (SES), we used analytical results based on the hypergeometric distribution to find the expectation and variance of the NODF statistic under both models. SES values were generally very large (>2); we used Wald tests to compute approximate *P* values.

### Environment distribution of taxa and Shannon entropy

For each Deblur tag sequence *B*, sample *s* in the set of all EMP samples *S*, and sample type (EMPO level 3) category *E*, defineas the fraction of total appearances of tag sequence *B* in sample type category *E* (with *N* possible values). For a given cluster of tag sequences *T* (phylogenetic subtree or taxonomic group, for example, Firmicutes), we then calculate cluster distribution vector aswhere *W*_*E*_ combined for all tag sequences in the sequence cluster is given byClusters of tag sequences were defined in two ways: first, by partitioning using the taxonomic lineage information for the tag sequences; second, by maximum tip-to-tip branch length for nodes on the phylogenetic tree. To calculate entropy of environment distribution as a function of taxonomic level (for example, phylum), the mean of Shannon entropies for all taxonomic groups belonging to that taxonomic level was calculated (weighted by the number of tag sequences in each taxonomic group). To calculate the entropy as a function of the phylogenetic subtree group width, cluster Shannon entropy was calculated for all subtrees, as well as the maximum tip-to-tip distance for each subtree. To ascertain whether changes in entropy between taxonomic and phylogenetic levels were expected given the observed distribution of environment entropy among tag sequences, a null model was calculated by randomly permuting the Deblur tag sequence taxonomy associations (for the entropy versus taxonomy analysis) or the phylogenetic tip placement (for the entropy versus phylogeny analysis). To reduce the effect of discretization on the entropy calculation in both analyses, clusters of tag sequences were included in the analysis only if they had a minimum of 20 tag sequences. For unique tag sequences (that is, a branch length threshold of 0.0), sequences were required to be found in a minimum of 10 samples. To calculate the approximate branch length corresponding to each taxonomic level, we found the lowest common ancestor for each group and calculated the maximum tip-to-tip distance in that subtree.

### EMP trading cards

We started with a BIOM table of 90-bp Deblur tag sequences (16S rRNA gene, V4 region), rarefied to 5,000 observations per sample, containing 2,000 samples evenly distributed across environments and studies (Extended Data Fig. 7a). From this we calculated the following: the number, fraction, and rank of samples in which a tag sequence is found; the abundance, fraction, and rank of observations represented by that tag sequence; the taxonomy of the tag sequence from Greengenes; and the list of all the samples in which the tag sequence is found. This summary is located at ftp://ftp.microbio.me/emp/release1/otu_distributions/. Additionally, for each tag sequence with a trading card in Extended Data Fig. 7b or http://www.earthmicrobiome.org/trading-cards, we identified sequences in RDP (http://rdp.cme.msu.edu)^82^ matching 100% along the 90-bp region of the 16S rRNA gene. Trading cards at http://www.earthmicrobiome.org/trading-cards are those with prevalence or abundance in the top 10 of all tag sequences or the most abundant tag sequence for each environment having a distribution Shannon entropy < 1, a proportion of that environment ≥ 25%, and total observations ≥ 1,000.

### Redbiom database service

A metadata and feature search service containing the EMP data is available through Redbiom. Redbiom is a caching layer for BIOM table and sample metadata, where by default it allows users to interact with the public portion of Qiita (which includes all of the EMP studies). This service allows users to find samples on the basis of sample details (for example, all soil samples with pH < 7), to find samples on the basis of features they contain (for example, all samples in which Greengenes ID 131337 exists), to find features on the basis of taxonomy (for example, all samples in which genus *Pyrobaculum* exists), to extract sample data into BIOM tables, and to extract sample metadata. Installation of the command-line client and usage instructions are available at https://pypi.python.org/pypi/redbiom; examples of command-line queries are provided at https://github.com/biocore/redbiom. A graphical user interface for Redbiom is available at http://qiita.microbio.me.

### Code availability

Code for reproducing sequence processing, data analysis, and figure generation is provided at https://github.com/biocore/emp and is archived at https://zenodo.org with DOI 10.5281/zenodo.1009693. Redbiom code is available at https://github.com/biocore/redbiom and is archived at https://zenodo.org with DOI 10.5281/zenodo.1009150.

### Data availability

Per-study sequence files and sample metadata are available from EBI (http://www.ebi.ac.uk/ena) with accession numbers in Supplementary Table 1. Per-study sequence files, sample metadata, and observation tables and information are available from Qiita (http://qiita.microbio.me) using the study IDs in Supplementary Table 1. EMP-wide sample metadata, observation tables and information (trees and taxonomies), alpha- and beta-diversity results, and observation summaries for trading cards are available at ftp://ftp.microbio.me/emp/release1; these files plus the Redbiom database at time of publication are archived at https://zenodo.org with DOI 10.5281/zenodo.890000.

## Supplementary information

Life Sciences Reporting Summary (PDF 72 kb)

Supplementary Table 1 This file contains Table 1 - **Studies within the Earth Microbiome Project included in this meta-analysis**. These studies constitute EMP 16S Release 1. Read length is median bp for sequences in that study. DOIs with an asterisk describe that study’s samples but did not use EMP sequence data. Sample counts exclude controls. (XLSX 91 kb)

Supplementary Table 2 This file contains Table 2 - **Description of metadata fields in the mapping files**. Also shown is data type (Python/Pandas), variable type, and number of the 27,751 samples having metadata for each field. (XLSX 82 kb)

Supplementary Table 3 This file contains Table 3 - **Top tag sequences by prevalence, abundance, and specialization for habitat**. The most prevalent (number of samples observed in), abundant (number of total times observed), or specific for a habitat (most abundant tag sequence with prevalence >25% of that habitat and Shannon entropy <1) EMP tag sequences. Tag sequences are 90-bp 16S rRNA gene sequences (V4 region, starting after primer 515f) from the Deblur algorithm. Values are for a subset of the EMP consisting of 2,000 samples with even representation across habitats (EMPO level 3) and studies, except for statistics on blanks, which are for all blank samples in the EMP (values greater in blanks than subset_2k are coloured red). Samples were rarefied to 5,000 observations per sample. tag sequences are sorted by prevalence. Sequences annotated as chloroplast were excluded before statistics were computed. (XLSX 47 kb)

## Related audio

Janet Jansson explains the ambitious goal to sequence Earth’s microbiome

## PowerPoint slides

PowerPoint slide for Fig. 1
PowerPoint slide for Fig. 2
PowerPoint slide for Fig. 3
PowerPoint slide for Fig. 4

## Source data

Source data to Fig. 1
Source data to Fig. 2
Source data to Fig. 3
Source data to Fig. 4
