# Supplementary material for: A communal catalogue reveals Earth’s multiscale microbial diversity
Source: Nature. 2017 Nov 1;551(7681):457–63. doi: 10.1038/nature24621 (PMC6192678; doi:10.1038/nature24621)
Supplement: Supplementary file 1 — Life Sciences Reporting Summary (PDF 72 kb) [file 41586_2017_BFnature24621_MOESM1_ESM.pdf]

nature research

Corresponding author(s): Thompson, Luke R. (submitting author)

☐ Initial submission ☐ Revised version ☒ Final submission

Life Sciences Reporting Summary

Nature Research wishes to improve the reproducibility of the work that we publish. This form is intended for publication with all accepted life science papers and provides structure for consistency and transparency in reporting. Every life science submission will use this form; some list items might not apply to an individual manuscript, but all fields must be completed for clarity.

For further information on the points included in this form, see Reporting Life Sciences Research. For further information on Nature Research policies, including our data availability policy, see Authors & Referees and the Editorial Policy Checklist.

Experimental design

1. Sample size

Describe how sample size was determined.

Two subsets of the EMP dataset were used for analyses presented in this paper. Fo r analysis of total diversity across the dataset (alpha- and beta-diversity in Figs. 2-3), we used the full set of 24,910 samples that passed minimal quality controls (QC-filtered) as described in the methods. For Figs. 4-6 and supplementary figures as noted, we used a 2000-sample subset containing samples picked randomly and evenly across 17 habitats a nd then evenly across studies in each sample type.

2. Data exclusions

Describe any data exclusions.

To generate the QC-filtered subset, samples were removed if they had fewer than a predetermined number of observations in the OTU/Deblur tables (see methods). Study no. 1799 was excluded from the QC-filtered subset because of concerns about contamination. For the effect size calculation (ED Fig. 5), categories within each predictor had a minimum of 75 samples per category, and predictors with values for less than half of samples were excluded. For ED Table 3, sequences annotated as chloroplast were excluded before statistics were computed.

3. Replication

Describe whether the experimental findings were reliably reproduced.

The experimental findings were reliably reproduced. For the purposes of this meta-analysis, having multiple samples from multiple studies for each habitat type constituted replication. Many studies within the meta-analysis had dedicated biological replicates. Nestedness results were reproduced using 5 additional randomly-selected 2000-sample subsets.

4. Randomization

Describe how samples/organisms/participants were allocated into experimental groups.

For creating subsets of samples, samples were drawn randomly, evenly across habitat types and studies. Results were reproduced using 5 additional randomly-selected 2000-sample subsets.

5. Blinding

Describe whether the investigators were blinded to group allocation during data collection and/or analysis.

Investigators were blinded; allocation to groups (subsets) was done entirely computationally and randomly.

Note: all studies involving animals and/or human research participants must disclose whether blinding and randomization were used.

6. Statistical parameters

For all figures and tables that use statistical methods, confirm that the following items are present in relevant figure legends (or in the Methods section if additional space is needed).

n/a	Confirmed	
☐	☒	The exact sample size (*n*) for each experimental group/condition, given as a discrete number and unit of measurement (animals, litters, cultures, etc.)
☐	☒	A description of how samples were collected, noting whether measurements were taken from distinct samples or whether the same sample was measured repeatedly
☐	☒	A statement indicating how many times each experiment was replicated
☐	☒	The statistical test(s) used and whether they are one- or two-sided (note: only common tests should be described solely by name; more complex techniques should be described in the Methods section)
☐	☒	A description of any assumptions or corrections, such as an adjustment for multiple comparisons
☐	☒	The test results (e.g. *P* values) given as exact values whenever possible and with confidence intervals noted
☐	☒	A clear description of statistics including central tendency (e.g. median, mean) and variation (e.g. standard deviation, interquartile range)
☐	☒	Clearly defined error bars

See the web collection on statistics for biologists for further resources and guidance.

Software

Policy information about availability of computer code

7. Software

Describe the software used to analyze the data in this study.	Code for reproducing sequence processing, data analysis, and figure generation is provided at github.com/biocore/emp and is archived at zenodo.org with DOI 10.5281/zenodo.XXXXXX. Redbiom code is available at github.com/biocore/redbiom and is archived at zenodo.org with DOI 10.5281/zenodo.XXXXXX. (Zenodo DOIs will be provided in proof stage, as discussed with the editor.)

For manuscripts utilizing custom algorithms or software that are central to the paper but not yet described in the published literature, software must be made available to editors and reviewers upon request. We strongly encourage code deposition in a community repository (e.g. GitHub). *Nature Methods* guidance for providing algorithms and software for publication provides further information on this topic.

Materials and reagents

Policy information about availability of materials

8. Materials availability

Indicate whether there are restrictions on availability of unique materials or if these materials are only available for distribution by a for-profit company.	No unique materials were used.

9. Antibodies

Describe the antibodies used and how they were validated for use in the system under study (i.e. assay and species).	No antibodies were used.

10. Eukaryotic cell lines

a. State the source of each eukaryotic cell line used.	No eukaryotic cell lines were used.
b. Describe the method of cell line authentication used.	No eukaryotic cell lines were used.
c. Report whether the cell lines were tested for mycoplasma contamination.	No eukaryotic cell lines were used.
d. If any of the cell lines used are listed in the database of commonly misidentified cell lines maintained by ICLAC, provide a scientific rationale for their use.	No commonly misidentified cell lines were used.

Animals and human research participants

Policy information about studies involving animals; when reporting animal research, follow the ARRIVE guidelines

11. Description of research animals

Provide details on animals and/or animal-derived materials used in the study.	Animal subjects are described in the original studies where animal-associated samples were collected. IACUC protocol numbers can be provided if necessary.

Policy information about studies involving human research participants

12. Description of human research participants

Describe the covariate-relevant population characteristics of the human research participants.	Human subjects are described in the original studies where human-associated samples were collected. IRB protocol numbers can be provided if necessary.
