# Supplementary figures and images for: A communal catalogue reveals Earth’s multiscale microbial diversity
Source: Nature. 2017 Nov 1;551(7681):457–63. doi: 10.1038/nature24621 (PMC6192678; doi:10.1038/nature24621)

## Slide 1
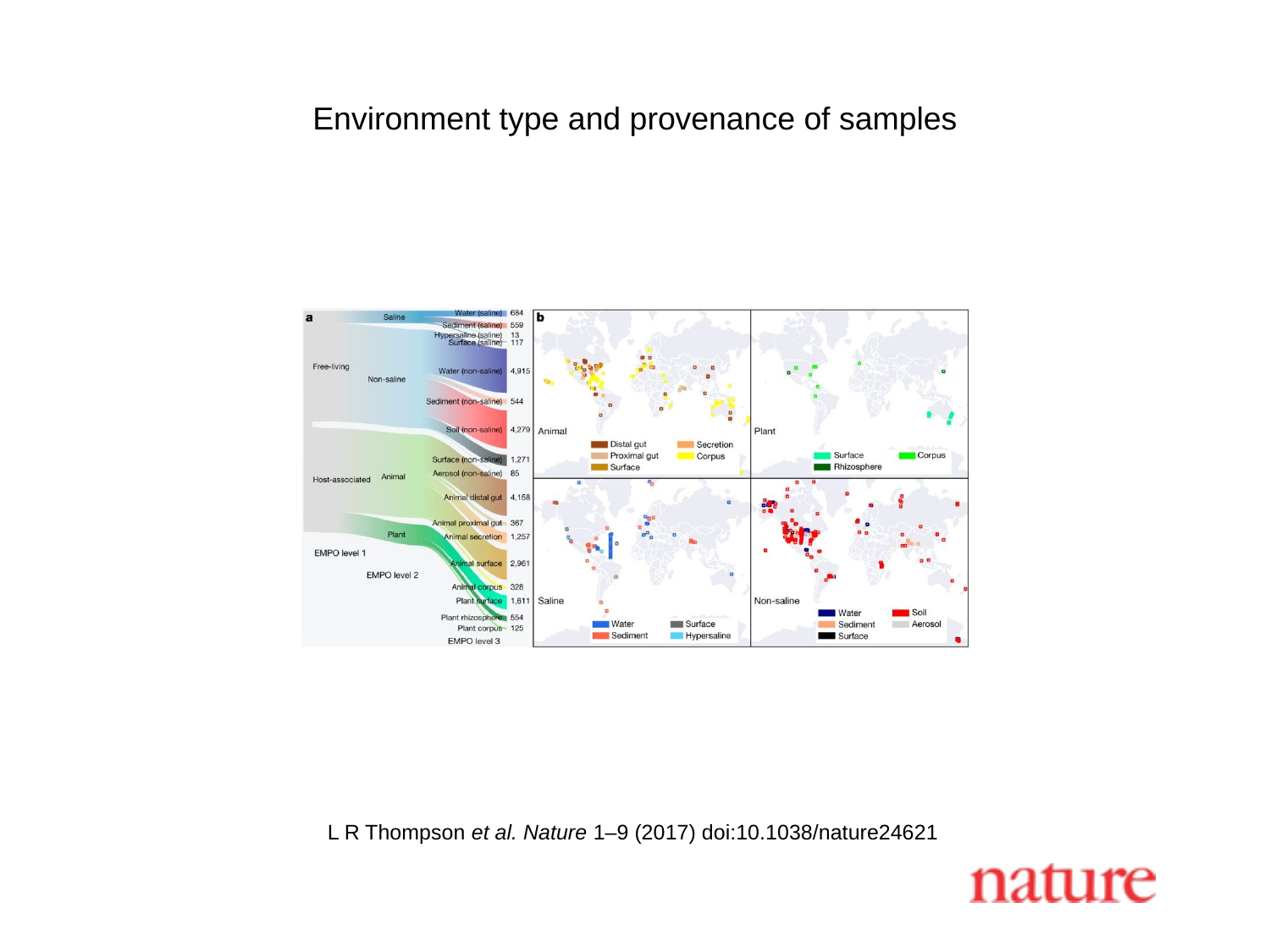

# Environment type and provenance of samples
L R Thompson et al. Nature 1–9 (2017) doi:10.1038/nature24621

Supplement: Supplementary file 6 — PowerPoint slide for Fig. 1 [file 41586_2017_BFnature24621_MOESM5_ESM.ppt]

## Slide 1
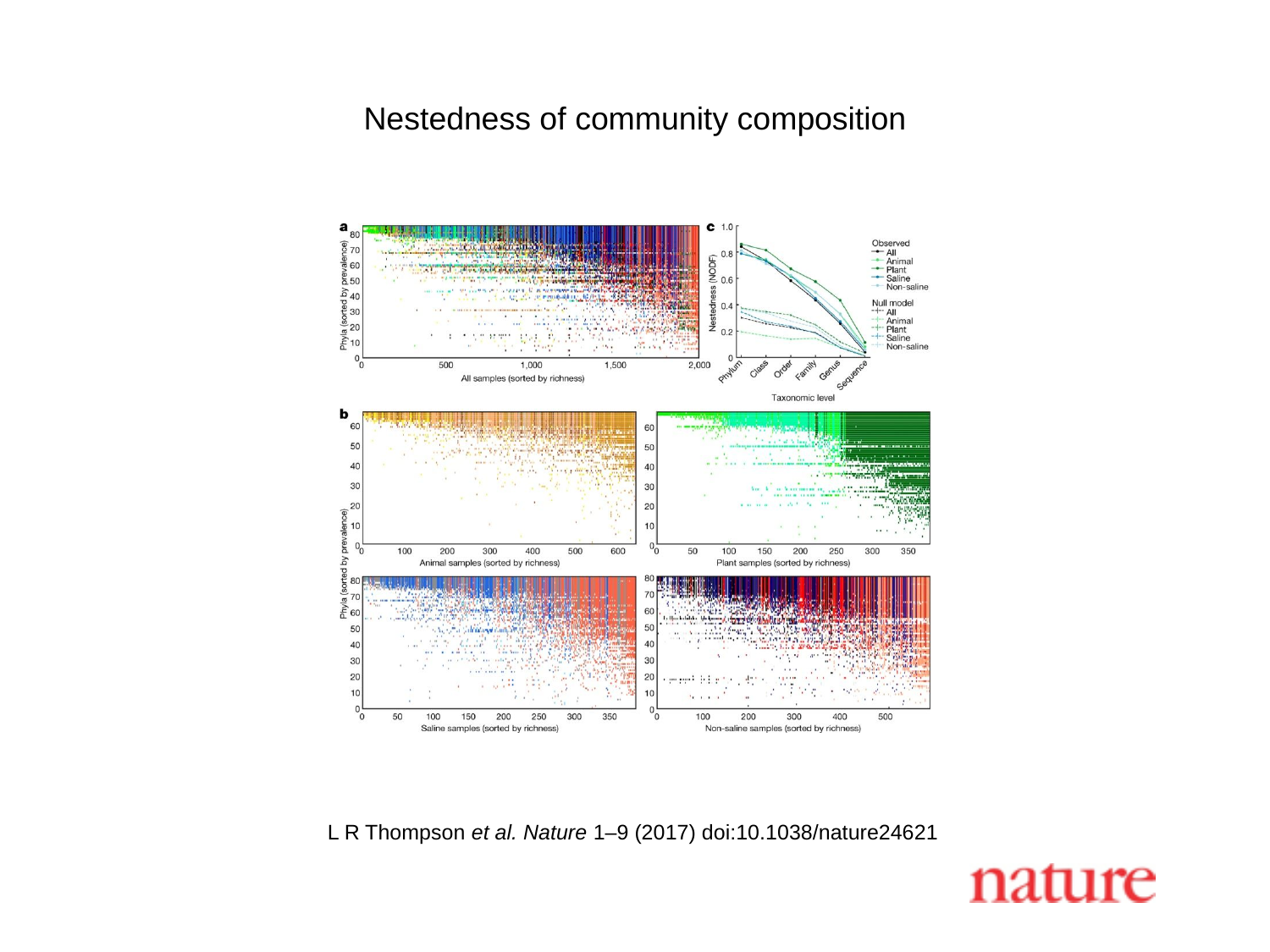

# Nestedness of community composition
L R Thompson et al. Nature 1–9 (2017) doi:10.1038/nature24621

Supplement: Supplementary file 8 — PowerPoint slide for Fig. 3 [file 41586_2017_BFnature24621_MOESM7_ESM.ppt]
