# Supplementary material for: A communal catalogue reveals Earth’s multiscale microbial diversity
Source: Nature. 2017 Nov 1;551(7681):457–63. doi: 10.1038/nature24621 (PMC6192678; doi:10.1038/nature24621)
Supplement: Supplementary file 7 — PowerPoint slide for Fig. 2 [file 41586_2017_BFnature24621_MOESM6_ESM.ppt]

## Slide 1
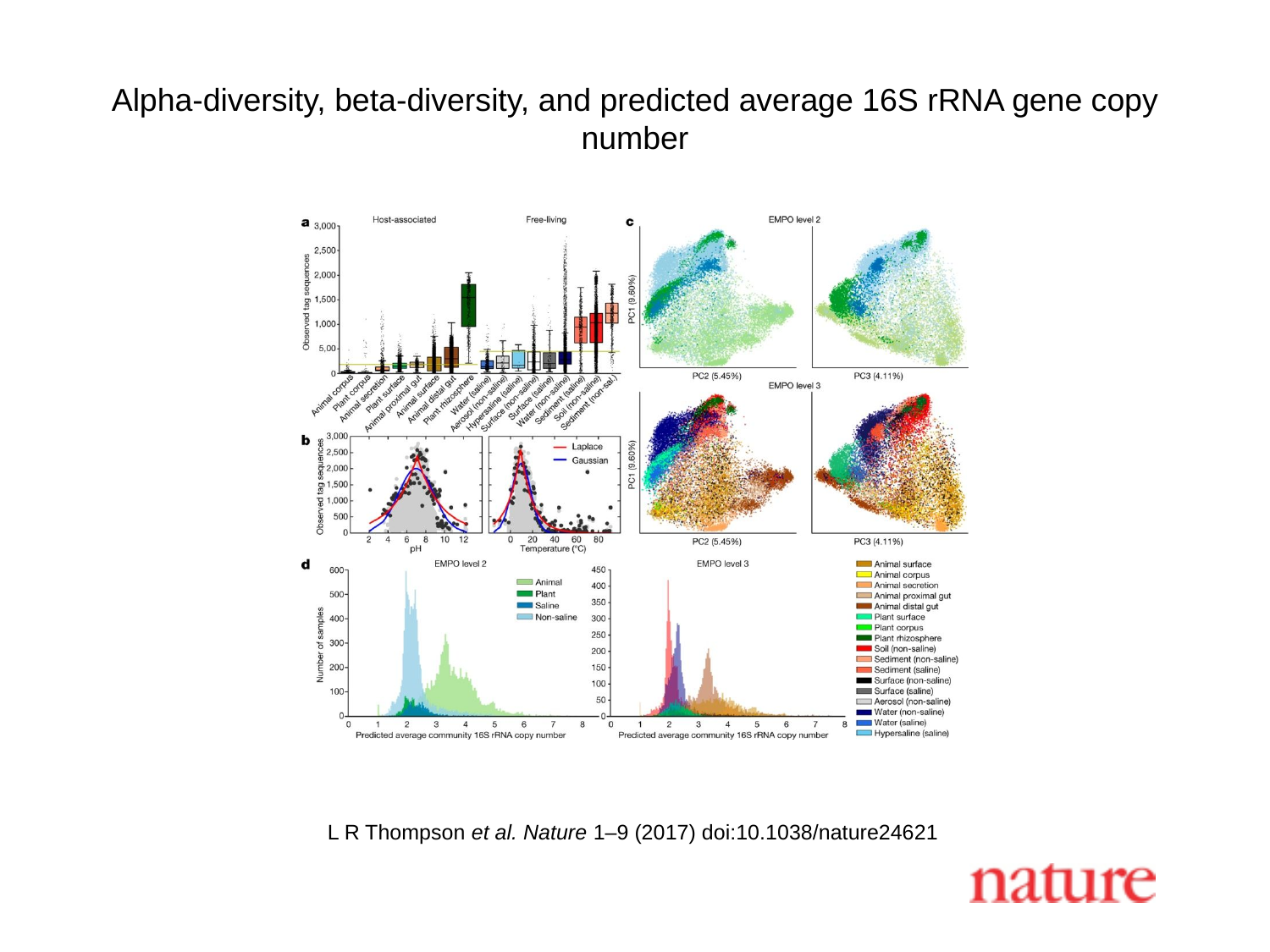

# Alpha-diversity, beta-diversity, and predicted average 16S rRNA gene copy number
L R Thompson et al. Nature 1–9 (2017) doi:10.1038/nature24621
